# Repeatability of radiomics studies in colorectal cancer: a systematic review

**DOI:** 10.1186/s12876-023-02743-1

**Published:** 2023-04-14

**Authors:** Ying Liu, Xiaoqin Wei, Xu Feng, Yan Liu, Guiling Feng, Yong Du

**Affiliations:** 1 School of Medical Imaging, North Sichuan Medical College, Sichuan Province, Nanchong City, 637000 China; 2 Department of Radiology, the Affiliated Hospital of North Sichuan Medical College, 1 Maoyuannan Road, Sichuan Province 637000 Nanchong City, China

**Keywords:** Colorectal cancer, Artificial intelligence, Radiomics, Repeatability, Machine learning

## Abstract

**Background:**

Recently, radiomics has been widely used in colorectal cancer, but many variable factors affect the repeatability of radiomics research. This review aims to analyze the repeatability of radiomics studies in colorectal cancer and to evaluate the current status of radiomics in the field of colorectal cancer.

**Methods:**

The included studies in this review by searching from the PubMed and Embase databases. Then each study in our review was evaluated using the Radiomics Quality Score (RQS). We analyzed the factors that may affect the repeatability in the radiomics workflow and discussed the repeatability of the included studies.

**Results:**

A total of 188 studies was included in this review, of which only two (2/188, 1.06%) studies controlled the influence of individual factors. In addition, the median score of RQS was 11 (out of 36), range-1 to 27.

**Conclusions:**

The RQS score was moderately low, and most studies did not consider the repeatability of radiomics features, especially in terms of Intra-individual, scanners, and scanning parameters. To improve the generalization of the radiomics model, it is necessary to further control the variable factors of repeatability.

**Supplementary Information:**

The online version contains supplementary material available at 10.1186/s12876-023-02743-1.

## Background

Colorectal cancer (CRC) is one of the most common clinical malignant tumors [1]. Medical imaging tools have become crucial in CRC for staging and treatment evaluation [2]. However, traditional radiology is mainly dependent on the subjective qualitative interpretations of the doctor [2], which often leads to suboptimal positive and negative predictive values [2, 3]. In recent years, with the rapid development of image analysis methods and pattern recognition tools, there is a growing shift away from qualitative to quantitative analysis of medical images [2].

As a quantitative analysis tool, radiomics extracts features from medical images through high-throughput computing and applies them to personalized clinical decisions to improve the accuracy of diagnosis and prognosis [4]. In recent years, radiomics showed a unique advantage for staging, differential diagnosis, and prognosis [5]. Although an increasing amount of radiomics research has been published, the comparability and repeatability of radiomics models remain a great challenge due to the lack of standardization in the field of radiomics [6, 7]. Assessing the repeatability of radiomics is necessary to achieve the clinical implementation of radiomics results and to ensure a high predictive capability of the radiomics model for a variety of populations and institutions [8]. In addition, several factors that affect the repeatability have been identified in the complicated workflow of radiomics, such as scanner [9–11], acquisition parameters [11–16], pretreatment method [17, 18], segmentation method [19–22], inter/intra-observer variability [16, 17, 19], feature selection method [23], modeling method [23].

Therefore, we conducted a systematic review to survey the repeatability of radiomics research in CRC. Furthermore, we gave some suggestions to increase radiomics repeatability for future research.

## Methods

### Review strategy

We conducted a systematic review according to the Preferred Reporting items for Systematic review and Meta-Analysis (PRISMA) checklist [24]. But the review was not registered before. The systematic search was conducted by two reviewers via PubMed and Embase databases until Jul 4, 2022. The full search strategies from Additional Text 1.

### Study selection

#### Population

We included primary research assessing the role of radiomics for diagnostic or prognostic with CRC patients. However, studies consisting of animal subjects and other types of articles than original articles (reviews, case reports, brief communications, technical reports, letters to editors, comments, and conference proceedings) were excluded.

#### Intervention

To be included, studies had to use the radiomics analysis of the preoperative or postoperative medical images in CRC patients for stratification of the CRC, prediction of response to therapy, or prognosis.

#### Outcome

In this review, the primary outcome of interest was the repeatability in the whole process of the radiomics research (including intra-individual, imaging acquisition, segmentation, feature selection, modeling, and evaluation). Studies with insufficient information for assessing the methodological quality were excluded.

#### Study extraction and quality assessment

The following data from each eligible study was systematically recorded: author, year, purpose, type, sample size, imaging modality, acquisition parameters, reconstruction parameters, pretreatment method, feature selection method, modeling method, segmentation method, number of features, verification method, performance index, and clinical utility.

In addition, the methodological quality of the eligible studies was assessed by the Radiomics Quality Score (RQS) [4]. The RQS was a unique quality assessment tool in radiomics [25], which score was composed of 16 parts with a total score of 36. A higher score represents better quality of the article. There were great differences in the methods used in the eligible studies, so the meta-analysis did not conduct.

#### Risk of bias in individual studies

The common bias analysis tools were not applicable here for the following reasons. First, the systematic review aims to assess the repeatability of radiomics research rather than the clinical purpose and outcomes. Second, there is no strictly causal association between repeatability and outcomes (diagnostic or prognosis performance). So the TRIPOD (Transparent Reporting of a multivariable prediction model for Individual Prognosis Or Diagnosis) and ROBINS (Risk Of Bias in Non-randomized Studies) were not applicable. Finally, the purposes of the eligible studies were highly heterogeneous, including staging, diagnosis, prognosis, and evaluating treatment. Thus, QUADAS-2(Quality Assessment of Diagnostic Accuracy Studies), which assesses the risk of bias in diagnostic studies, and QUIPS (Quality in Prognosis Studies), which assesses the risk of bias in prognostic studies, were not applicable.

Quality assessment was conducted using the RQS. Furthermore, the risk of bias in the eligible studies was assessed by two reviewers from the following specific aspects:

1. Sufficiency of method description and disclosure (imaging acquisition, stability of the segmentation, details of the selected features, methods for selecting features and modeling, and sufficiency of model performance description).
2. Description of the code used to compute features, establish models, verify models, and statistical analysis.
3. The methods to improve study reproducibility (phantom study and test–retest study) and validating the model in the external validation cohort.

## Results

### Study selection

**Fig. 1:**
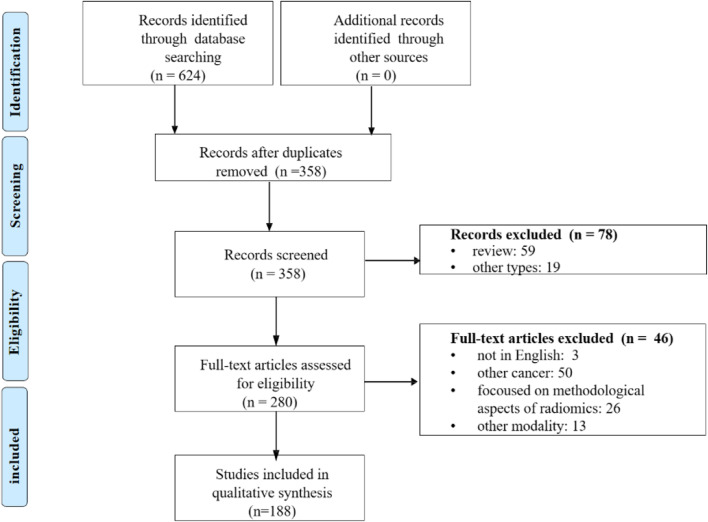
Flow diagram of study selection process

A total of 624 articles were retrieved from the comprehensive literature search (PubMed and Embase), of which were reduced to 358 based on screening of title and abstract. Of these studies, 188 articles were included after the full-text articles were assessed for eligibility. Include and exclude flowchart is shown in Fig. 1.

### Statistics of the studies

The current situation of radiomics in patients with colorectal cancer was analyzed in the following aspects, and the included articles were given an overall overview. The key information of included studies is summarized in Additional Table 1.

**Fig. 2:**
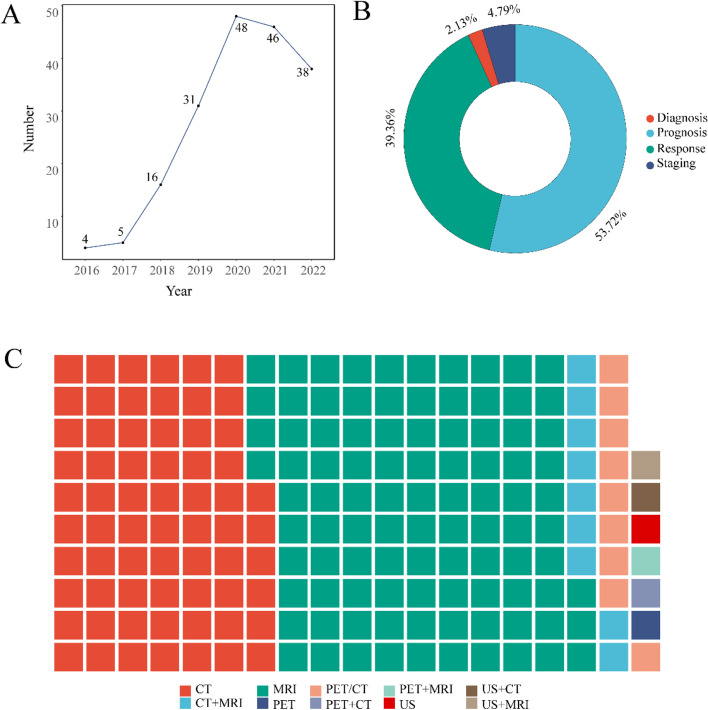
General situation of radiomics studies. **A**, Published frequency of radiomics studies on colorectal cancer from 2016 to 2022; **B**, Pie chart of the research purpose of studies; **C**, waffle plot of imaging mode of studies

Figure 2A shows the published number based on radiomics in patients with colorectal cancer in recent years. The number of articles continued to increase from 2016 to 2020 and decreased from 2020 to 2022. The publication number has an overall increasing trend.

The included studies were designed to assess the prognosis (101, 53.72%), response (74, 39.36%), staging (9, 4.79%), and diagnosis (4, 2.13%) of patients with colorectal cancer (Fig. 2B). Predictive performance was moderate to good in all radiomics models, but performance varied widely between models (the area under the curve values ranging from 0.56 to 0.98).

The imaging modalities of the studies include CT, MRI, PET, PET-CT, US, and multi-modality (Fig. 2C). It is worth noting that CT and MRI account for 35.11% (66/188) and 51.60% (97/188) of all studies.

Most radiomics of colorectal cancer were based on retrospective data sets (177, 94.15%), and only a few were prospective studies. Multicenter studies might fully reflect the overall situation and evaluate the generalization ability of the model. Of all the studies, only 9.04% (17/188) of articles had data from multiple medical institutions and 12.77% (24/188) of articles were dual-center studies.

### Quality of the studies

#### RQS score

The included articles were scored by RQS, and the specific scores were shown in Additional Table 2. Its score ranges from-1 to 27 (-3.03% to 75.00%), the median was 11 (30.56%), of which 93.09% (175) studies scored less than 50%.

**Fig. 3:**
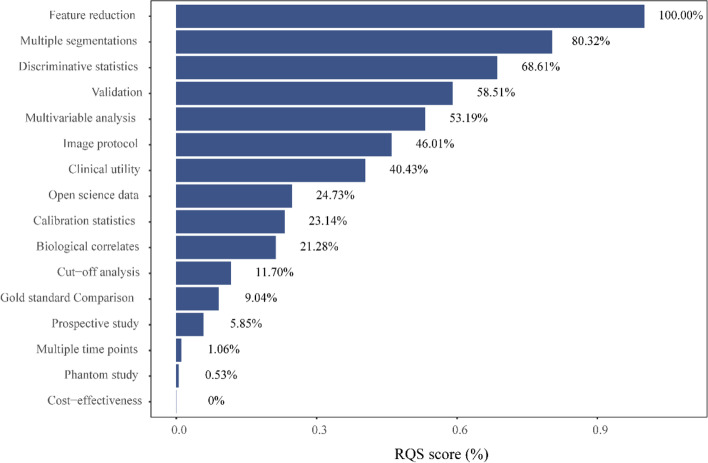
Completion rate of 188 studies in RQS

The total score of each item in the RQS is different, so it is difficult to compare the scores of each item. Therefore, the score ratio (actual score /total score*100%) was used to compare the scores of each item (Fig. 3).

Even though feature reduction was performed in all studies, 34.57% (65/188) of all studies still had the risk of overfitting, following the “one in ten” rule of thumb (at least ten patients for each feature in the model) [26]. Although well-documented image protocols for the studies were provided in 173 articles, only P Lovinfosse, et al. [27] showed [8] their study’s images were acquired and reconstructed according to the public image protocols. The cost-effectiveness of the clinical application has not been considered in the included studies, which is one of the most important components for the clinical application of the models. Only 0.53% (1/188) of the studies detect inter-scanner differences by phantom (Phantom study), and only 1.06% (2/188) analyzed feature robustness to temporal variabilities (Multiple time points).

#### Risk of bias

The potential risk of bias was present in the included studies to varying degrees and different aspects. The risk of bias in the studies was assessed in the terms of method details provided, code details provided, and repeatability (Additional Table 3).

#### Repeatability

**Fig. 4:**
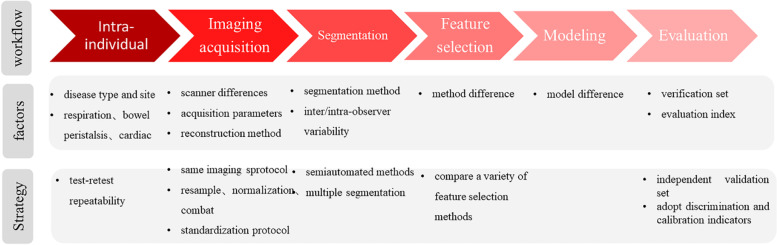
Radiomics workflow and repeatability. Each step has associated factors which may influence the repeatability of the study. Although modelling affect reproducibility, there is still no solution

The repeatability of radiomics features was directly related to the accuracy of model [11]. The repeatability might be affected by many factors in the radiomics process, Such as scanner [9–11], acquisition parameters [11–16], pretreatment method [17, 18], segmentation method [19–22], inter/intra-observer variability [16, 17, 19], feature selection method [23], modeling method [23]. The factors and solutions in the radiomics workflow are shown in Fig. 4.

#### Intra-individual repeatability

Radiomic features may be influenced by organ motion or expansion or shrinkage of the target volume caused by physiological factors such as respiration, bowel peristalsis, cardiac and cardiac activity [4]. The robustness of the radiomics features affected by various physiological factors of the individual was called intra-individual repeatability. The intra-individual repeatability was neglected in the majority of included studies, while the robustness of the features was analyzed by test–retest in only two (2/188, 1.06%) studies [28, 29]. X Ma, et al. [28] set the basis of an intraclass correlation coefficient (ICC) of 0.6 for test–retest analysis, but the population or phantom, time interval, and the ICC form in the test–retest analysis were not described. J Wang, et al. [29] performed a test–retest analysis on 40 patients with stage II rectal cancer, which scanned twice using the same scanner and imaging protocol before treatment, and calculated the Spearman’s correction coefficients for each feature. Although the test–retest analyses were taken, the methodology among the two studies varied considerably.

#### Acquisition parameters

The radiomics features were derived from the same scanner and imaging protocol in the 89 (89/188,47.34%) studies, and the impact on radiomics features due to scanner differences might be reduced. Notable was that K Nie, et al. [30] underwent quality assurance checks monthly and maintained bimonthly to ensure the image quality. And the standardized uptake value (SUV) measurement difference between the two scanners was reduced to less than 10 percent through regular standardization and quality assurance in the study of J Kang, et al. [31]. The effect of acquisition parameters on radiomics features might be further controlled by taking the above measures.

In addition, various image post-processing approaches were conducted to reduce the variation of the radiomics features. The image intensity discretization and normalization were investigated to reduce the noise and inconsistencies in the 60 (60/188, 31.91%) studies. In total, 42 (42/188, 22.34%) studies conducted the resampling, which was performed by linear interpolation, to mitigate the influence of the layer thickness. The size of voxels after resampling was not uniform between the included studies.

#### Segmentation

The radiomics features' robustness may be related to the segmentation of regions of interest (ROI) [10]. The segmentation method includes manual, semi-automatic, and automatic segmentation. Manual segmentation is usually regarded as the gold standard, but it was time-consuming [19] and suffered from great subjective differences among the observers [32, 33].

The advantages of semi-automatic segmentation in terms of segmentation time have been reported in the existing studies [19–21, 34, 35]. MM van Heeswijk, et al. [19] analyzed the segmentation methods and found that semi-automatic segmentation has similar accuracy to manual segmentation while reducing the time by 4 min (manual segmentation time 60–1118 s). Semi-automatic segmentations were used in 22 (11.70%) studies, while automatic segmentations were used in 11 (5.85%) studies. The segmentation methods were not mentioned in the twelve (6.38%) studies, manual segmentations were adopted in the remaining 143 (76.06%).

The radiomics features might be affected by segmentation variation (inter-observer variation and intra-observer variation), while multi-person/multi-method segmentation may reduce segmentation variation [4]. The multi-person/multi-method segmentation was not performed in 37 (37/188, 19.68%) studies. Segmentation variation was not measured quantitatively in 44 studies but was measured with different parameters in the remaining 111 studies. Among the included studies, intraclass correlation coefficient (ICC), Dice similarity Coefficient and/or Jaccard similarity coefficient, Bland–Altman plots, and Spearman correlation coefficient was used as evaluation parameters in 94 (94/111, 84.68%), 9 (9/111,8.11%), 1 (1/111, 0.90%), and 1 (1/111, 0.90%) articles, respectively. Notable was that P Lovinfosse, et al. [27] used automatic segmentation which repeatability was verified, and 4 articles [26, 36–38] defined a segmentation z-score that captures the robust features in the segmentation. In a word, most studies (84.68%) used ICC to evaluate segmentation variability, while I Fotina, et al. [39] preferred to use the Jaccard similarity coefficient, conformal number, or generalized conformability index to evaluate segmentation variability.

The ROI definition among studies varied considerably. The tumor region was defined as the ROI in most studies (174/188, 92.55%), while the peri-tumor region was also taken into account in 6 (6/188, 3.19%) studies. Three (3/188, 1.60%) studies used the entire parenchyma as the ROI. In addition, the tumor region and the lymph node region were defined as the ROIs in 3 (3/188, 1.60%) studies, and the ROIs were traced manually along the largest lateral pelvic lymph node in the study of R Nakanishi, et al. [40].

#### Feature selection

A large number of features were extracted in the radiomics studies, leading to the dimension disaster and the model overfitting so greatly reducing the generalization ability of the model [41, 42]. To reduce the false positive rate, A Chalkidou, et al. [42] proposed the following measures: (1) repeatability of features (2) cross-correlation analysis (3) inclusion of clinically important features (4) at least 10–15 patients with each feature (5) external verification.

The main purposes of feature selection were (1) to select repeatable features among the institutions, (2) to remove redundant features (highly related features between features), and (3) to select features with predictive potential. Various methods and combinations of feature selection were applied to reduce the number of features [43]. In total, only one feature selection method was used in 77 (77/188, 40.96%) articles, while two, three, four, five, and six feature selection methods were used in combination in 62 (62/188, 32.98%), 36 (36/188, 19.15%), 11 (11/188, 5.85%), 1(1/188, 0.53%), and 1(1/188, 0.53%) articles, respectively. L Boldrini, et al. [44] extracted only two features to predict the clinical complete response after neoadjuvant radio-chemotherapy, so no feature selection measures were taken. The Least Absolute Shrinkage and Selection operator (LASSO) (106/188, 56.38%) was the most commonly used, followed by correlation analysis (52/188, 27.66%). The feature selection scheme should be adjusted according to the number of features and samples [41].

**Fig. 5:**
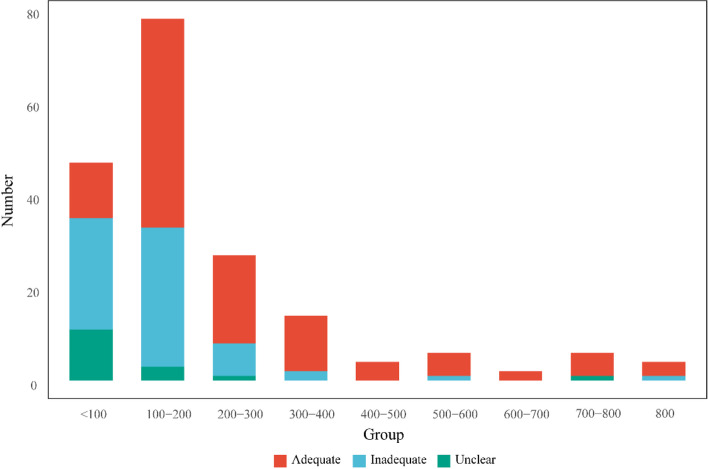
Sample size of included studies. Adequate sample means the ratio of the sample size to the feature number of the study is more than 10, inadequate sample means the ratio is less than 10, unclear means the study did not establish a model or did not specify the number of features

The sample size of all studies ranged from 15 to 918, with a median of 149, and 66.49% (125/188) of the studies had a sample size of 0–200. To assess the adequacy of the sample size in the study, MA Babyak [26] suggested that at least 10–15 patients were needed for each feature. Based on this standard, 65 (65/188, 34.57%) of the included studies did not meet the above conditions except 4 (4/188, 2.13%) studies [44–47] which did not establish a model, and 14 (14/188, 7.45%) studies did not indicate the number of features (Fig. 5).

#### Modelling methodology

Five (5/188, 2.66%) studies [27, 44–47] analyzed the predictive performance of individual radiomics features, while no predictive models were constructed. M Hotta, et al. [46] reported the gray-level co-occurrence matrix entropy was the relevant feature for overall survival and progression-free survival. The delta radiomics features (L_least and glnu) were the most predictive feature ratios in clinical complete response prediction in the studies of L Boldrini, et al. [44]. Subsequently, D Cusumano, et al. [45] validated the prognostic potential of these delta radiomics features on an external validation cohort, while the accuracy of L_least is significantly higher than glnu. AA Negreros-Osuna, et al. [47] found that the spatial scaling factor was the potential biomarker for determining BRAF mutation status and predicting the 5-year overall survival.

Radiomics models were constructed in 183 (183/188, 97.34%) studies, while prediction models were constructed by combining clinical factors with radiomics features in 100 (100/184, 54.35%) studies. Z Liu, et al. [48] constructed a radiomics-clinical model which significantly improved the classification accuracy compared to the clinical model, based on the integrated discrimination improvement values. And the radiomics model outperformed the qualitative analysis by radiologists in the study of H Tibermacine, et al. [36].

A variety of models including machine learning models and statistical models were used in the radiomics studies, such as logistic regression (LR), random forest (RF), supporter vector machine (SVM), K nearest neighbor (KNN), neural network (NN), Bayesian network (BN). The LR (96/183, 52.46%), SVM (20/183, 10.93%) and RF (20/183, 10.93%) were the frequently used models in the included studies. Noteworthy, SP Shayesteh, et al. [49] observed the ensemble model of machine learning classifiers (SVM.NN.BN.KNN) showed the best predictive performance. And Z Zhang, et al. [50] performed a retrospective study on 189 patients with locally advanced rectal cancer to assess the performance and stability of classification methods (SVM, KNN and RF), the result showed that the RF outperformed KNN and SVM in terms of AUCs.

#### Evaluation

According to the principle of confirmatory analysis, the generalization ability of the model could be evaluated in the independent verification set [51]. However, only 144 (144/188, 76.60%) articles used the independent dataset validation, the internal validation was performed by applying resampling methods in the remaining studies.

The performance of the models was assessed in the terms of discrimination, calibration, and clinical utility. The discrimination statistics (C-statistic, ROC curve and AUC) of the models were reported in 173 (173/188, 92.02%) studies, and resampling analysis (bootstrapping and cross-validation) were applied in 78 (78/173, 45.09%) studies. The calibration statistics (calibration curve or Brier score) of the models were mentioned in only75 (75/188, 39.89%) articles s, of which 12 (12/75, 16.00%) used resampling analysis. In addition, the clinical utility was evaluated by the decision curve in 76 (76/188, 40.43%). The differences in evaluation metrics between studies lead to difficulties in comparing performance between models.

### How to increase repeatability

#### Standardization protocol

D Mackin, et al. [9] performed a phantom analysis to compare the radiomics features extracted from four CT scanners (GE, Philips, Siemens, and Toshiba) and found that radiomics features might be affected by the scanners. The result was subsequently confirmed in the study of R Berenguer, et al. [11], who used two phantoms (the pelvic phantom and the phantom of different materials) to detect the features of intra-CT analysis (differences between different CT acquisition parameters) and inter-CT analysis(differences between five different scanners). R Berenguer, et al. [11] found that 71/177 were reproducible, and reported that the influence of scanners could be reduced by standardizing the acquisition parameters. RJ Gillies, et al. [37] have the same point of view.

The image Biomarker Standardization Initiative (IBSI) [52] standardized the definition, naming, and software of radiomics features. The Quantitative Imaging Network (QIN) [38] project initiated by NIC (National Cancer Institute) has promoted the standardization of imaging methods and imaging protocols. In addition, the Quantitative Imaging Biomarkers Alliance (QIBA) [53] organization sponsored by, the Radiological Society of North America (RSNA) has developed a standardized quantitative imaging document "Profiles" to promote clinical trials and practices of radiomics.

#### Test–retest reliability

JE van Timmeren, et al. [54] scanned forty patients with rectal cancer twice with the same scanning scheme at 15-min intervals and found that some radiomics features were not repeatable at different times for the same individual. But a set of highly reproducible radiomics features could be extracted by the test–retest based on phantom or patients [55, 56]. Moreover, JE van Timmeren, et al. [54] indicated that appropriate test–retest would be applied in the terms of the effects of hardware, acquisition, reconstruction, tumor segmentation, and feature extraction.

#### Post-processing

With the continuous emergence of new features, the efficiency of test–retest research becomes lower [18]. The variability of features might be reduced by the following post-processing methods.

Resampling and normalization: L He, et al. [15] demonstrated that acquisition parameters (slice thickness, convolution kernel, and enhancement) had affected the diagnostic performance of radiomics. Similarly, L Lu, et al. [14] demonstrated variation of radiomics features due to the slice thicknesses and reconstruction methods. Features associated with tumor size, border morphology, low-order density statistics, and coarse texture were more sensitive to variations in acquisition parameters. Subsequently, a more rigorous experiment [13] showed that 63/213 features were affected by voxels, but 42 features were significantly improved, and 21 features changed greatly after resampling. Therefore, some studies [12, 13, 57]showed that resampling could effectively improve the feature variation caused by voxel differences. However, resampling alone is not enough [13], normalization was used to reduce the influence of different gray ranges or the effects of low frequency and intensity inhomogeneity in some studies. Introducing noise, blurring the image, and causing the loss of image details are the disadvantages of normalization [58].

ComBat: Previously, genomics has been affected by batch effects, that is, systematic technical biases introduced by samples in different batches of processing and measurement that are not related to biological status [59]. WE Johnson, et al. [60] developed and validated a method to deal with the "batch effect"-ComBat. In radiomics, the impact of different scanners or scanning schemes is similar to the batches. Studies [61–63] showed that ComBat could reduce the feature differences caused by different scanners or scanning schemes, and retain the feature differences formed by biological variation. Although ComBat is practical, convenient and fast, it will be affected by the distribution of validated data sets [18], and it cannot be directly applied to imaging data [64]. So Y Li, et al. [18] developed a normalization method based on deep learning, which may effectively avoid the above problems.

## Discussion

This review analyzed the repeatability of radiomics on patients with colorectal cancer by discussing the method details in 188 studies and evaluated the quality of studies by RQS. Although the included studies demonstrated excellent predictive performance, the methodology varied considerably among studies in the terms of imaging parameters, feature selection and modeling, so the comparison between study results is difficult. In addition, the values of test–retest have been investigated for improving study reproducibility, but test–retest was rarely used in the included studies. In the review, many radiomics studies had poor quality by RQS.

Since 2016, the number of radiomics studies on patients with colorectal cancer continued to increase, but the low RQS score (-1 ~ 27) was shown in many studies, indicating that radiomics was an immature new technology in the field of colorectal cancer. This finding has been confirmed in the previous study [65]. C Xue, et al. [8] demonstrated the RQS is highly correlated with reliability, especially in the phantom study and imaging at multiple time points. P Lambin, et al. [4] explained that multiple time point was used to eliminate the unstable radiomics features which strongly related to organ movement or the expansion or shrinkage of the target volume, and reduce the influence of intra-individual variability [55, 56]. J Wang, et al. [29] selected the most stable radiomics features based on the test–retest on 40 patients with rectal cancer. Although the subjects of the test–retest could be patients or phantoms [66, 67], the unnecessary radiation damage might be increased in the patients’ test–retest. In addition, JE van Timmeren, et al. [54]proposed that extensive retest-test experiments can provide a stable set of radiomics features, and emphasized that retest-test studies should be adopted in each step, including intra-individual variability, scan acquisition and reconstruction, tumor segmentation and feature extraction. Open science data includes opening scanning information, opening segmentations of ROIs, opening source code and opening feature extraction methods (including formulas)[4]. Opening science data is beneficial for other researchers to reproduce the research results (independent researchers use the same technology and different data to repeat the research results and independently verify the results) and promote the application of the research model in clinical practice [3, 4, 6].

There are many variable factors and a wide variety of diseases in radiology, so the standardization of scanning parameters, acquisition parameters and reconstruction parameters could effectively reduce variability [3, 6]. In addition, the influence of scanning parameters might be reduced by using the same scanning scheme, unstable features may be reduced by retest tests [66], resampling might be used to control the influence of slicer thickness [13], and the "batch effect" could be reduced by combat method [61–63]. To balance the deviation of imaging features from four institutions, Z Liu, et al. [48] normalized the data, adopted combat methods to control the deviation of radiomics features, and weighted the radiomics features with inverse probability of treatment weighting (IPTW) to eliminate the covariant effect among the four cohorts. M Taghavi, et al. [68] conducted two experiments with the fake label to verify the validity of the radiomics and the results show that the model was not influenced by noise (first experiment) or the patient distribution across hospitals (second experiment).

For the method of segmentation, semi-automatic segmentation was not only more efficient but also useful to reduce the variability of manual segmentation [19–21, 34, 35]. Of all the studies, 143 (143/188, 76.06%) were segmented manually, and only 22 (22/188, 11.70%) were segmented semi-automatically. For inter-observer variability, only 111 studies took into account inter-observer variability and excluded instability. Even though the Jaccard index, Cn and CIgen may be better as evaluation parameters [39], 94 (94/111, 84.68%) studies were evaluated by ICC.

According to the Harrell criterion [69], the sample size should be more than 10 times the number of variables, and feature selection can reduce redundant features and reduce the risk of model over-fitting [41, 42]. Such as, random forest on 89 patients was used to measure the Gini importance of parameters, and finally 10 important parameters were included in the study of C Yang, et al. [70]. M Sollini, et al. [71] recommended that at least 50 patients would be included in the radiomics studies because the quality of the studies and the credibility of the results might be seriously affected by the sample size [72]. But, M Hennessy, et al. [73] constructed the formal model, which was the mathematical algorithms formulated by the experts. The formal model was more interpretable than machine learning models and does not require large amounts of data for validation[74]. The formal models have been applied and have shown excellent predictive performance in previous studies [74–79]. However, the formal methods were not widely used and the predictive performance of the models needed to be further validated in the field of cancer.

It is also important to avoid overly optimistic results in addition to repeatability. In many studies, the type I errors might be increased by the combination of optimal cut-off method and multiple hypothesis testing [42], while the multiple hypothesis test correction (Holm Bonferroni or Benjamin-Hochberg pair) was helpful to reduce class I errors [6].

According to the review of 188 studies, the lack of repeatability was the key problem in radiomics studies, and the standardization of radiomics processes helped comparing the existing studies [37]. It is difficult to form global standardization at present [37], but the repeatability of studies might be improved through open science data [4], retest-test research [66] and post-processing. The radiomics was used to predict the sequencing results by the DNA microarray in some studies [80–86]. Notable was that there were no studies on the combination of liquid biopsy and radiomics. Studies have shown that the combination of liquid biopsy and imaging could play an early warning role in patients prone to recurrence and metastasis [87]. In the future, it may be possible to add biomarkers from other disciplines to the radiomics model. T Cheng, et al. [88] proposed that a pattern consisting of three or more biomarkers could improve the accuracy and specificity of tumor prediction, diagnosis and prognosis, that is, pattern recognition [88]. Delta radiomics is to quote the time component in the study, that is, to use the image data of multiple time points for radiomics analysis. This method is expected to improve the diagnosis, prognosis prediction and treatment response evaluation [4]. Since most radiomics of colorectal cancer were based on retrospective data sets (177, 94.15%), more prospective studies with large samples and multiple centers are needed to promote the development of radiomics in the field of colorectal cancer. Cost-effectiveness analysis from an economic point of view is necessary for the clinical application of colorectal cancer imaging in the future [4].

The main limitations of this review are shown below. First, the meta-analysis was not conducted due to there being great differences in the methods of the included studies. Second, some related studies might not be retrieved, such as some grey literature. In this paper, only RQS is used to evaluate the quality of the article.

## Conclusion

Although existing studies showed that radiomics is helpful to the personalized treatment of patients in the field of colorectal cancer, there are still many challenges that remain to be solved. According to the RQS score, the quality of included studies was moderately low. Moreover, the main reason for the low RQS score was the lack of repeatability, most studies did not eliminate the influence of scanners, imaging parameters, and other factors. Therefore, these studies of lower quality and lack of repeatability mean that the results are not universal. In the future, larger samples and multicenter prospective high-quality studies are needed, and researches should focus on building a more stable and repeatable model.

## Supplementary Information

**Additional file 1. **

**Additional file 2: Table 1. **Study characteristics of 188 papers. **Table 2. **RQS of studies. **Table 3. **Risk of bias in individualstudies. 

## Data Availability

The datasets used and/or analysed during the current study are available from the corresponding author on reasonable request.

## Abbreviations

AUC: Area Under the Curve
CRC: Colorectal cancer
CRT: Chemoradiotherapy
CT: Computed Tomography
IBSI: Image Biomarker Standardization Initiative
ICC: Intraclass Correlation Coefficient
LASSO: Least Absolute Shrinkage and Selection operator
MRI: Magnetic Resonance Imaging
PET-CT: Fluorodeoxyglucose Positron Emission Tomography-Computed Tomography
PRISMA: Preferred Reporting items for Systematic review and Meta-Analysis
QIBA: Quantitative Imaging Biomarkers Alliance
QIN: Quantitative Imaging Network
RQS: Radiomics Quality Score
RSNA: Radiological Society of North America
SUV: Standardized Uptake Value
UA: Ultrasound
