# Supplementary material for: Repeatability of radiomics studies in colorectal cancer: a systematic review
Source: BMC Gastroenterol. 2023 Apr 14;23:125. doi: 10.1186/s12876-023-02743-1 (PMC10105401; doi:10.1186/s12876-023-02743-1)
Supplement: Supplementary file 1 — Additional file 1. [file 12876_2023_2743_MOESM1_ESM.docx]

**Additional Text**

We searched in the following electronic databases: MEDLINE/PubMed (National Center for Biotechnology Information, NCBI) and Embase for studies. We separately consulted the online database using a search string containing free-text and/or Medical Subject Headings (MeSH) search. The search strategy implemented in all databases is listed below:

**1.PubMed Search Strategy**

1.1 Available via https://pubmed.ncbi.nlm.nih.gov

1.2 Search date: Jul 4, 2022

1.3 Search strategy and result:

| **No.** | **Query** | **Result** |
| --- | --- | --- |
| **#1** | “Colorectal Neoplasms” [MeSH] | 226596 |
| **#2** | (“colon” [tiab] OR “rectal” [tiab] OR “colorect*” [tiab]) AND (“tumor*” [tiab] OR “cancer” [tiab] OR “neoplasm*” [tiab] OR “carcinoma*” [tiab]) | 270647 |
| **#3** | #1 OR #2 | 329124 |
| **#4** | “radiomic*” [tiab] OR “clinical-radiomics” [tiab] OR “radiomics analysis” [tiab] OR “radiomic* feature*” [tiab] OR “radiomic analysis” [tiab] | 6142 |
| **#5** | #3 AND #4 | 351 |
| **#6** | animals[mh] NOT humans[mh] | 5021368 |
| **#7** | #5 NOT #6 | 350 |

**2.** **Embase Search Strategy**

2.1 Available via www.embase.com

2.2 Search date: Jul 4, 2022

2.3 Search strategy and result:

| **No.** | **Query** | **Result** |
| --- | --- | --- |
| **#1** | exp colorectal tumor/ | 33617 |
| **#2** | ('colon'.ti,ab,kw. OR 'rectal'.ti,ab,kw. OR 'colorect*' .ti,ab,kw.) AND ('tumor*' .ti,ab,kw. OR 'cancer' .ti,ab,kw. OR 'neoplasm*' .ti,ab,kw. OR 'carcinoma*' .ti,ab,kw.) | 395963 |
| **#3** | #1 OR #2 | 402537 |
| **#4** | exp radiomics/ | 5024 |
| **#5** | 'radiomic*' .ti,ab,kw. OR 'clinical-radiomics' .ti,ab,kw. OR 'radiomic* analysis' .ti,ab,kw. OR 'radiomic* feature*' .ti,ab,kw. | 8610 |
| **#6** | #4 OR #5 | 8972 |
| **#7** | #3 AND #6 | 484 |
| **#8** | limit 7 to (human and english language and (article or article in press)) | 274 |

Duplicates were removed by using EndNote's duplicate identification strategy and then manually.
