# Supplementary material for: Repeatability of radiomics studies in colorectal cancer: a systematic review
Source: BMC Gastroenterol. 2023 Apr 14;23:125. doi: 10.1186/s12876-023-02743-1 (PMC10105401; doi:10.1186/s12876-023-02743-1)
Supplement: Supplementary file 2 — Additional file 2: Table 1. Study characteristics of 188 papers. Table 2. RQS of studies. Table 3. Risk of bias in individualstudies. [file 12876_2023_2743_MOESM2_ESM.docx]

**Additional Table 1: Study characteristics of 188 papers**

| **DOI/PMID** | **Aim** | **Design** | **Sample size** | **Imaging timing** | **Modality** | **Model** | **Result** |
| --- | --- | --- | --- | --- | --- | --- | --- |
| 10.3390/cancers12082027 | Response | Retrospective | T:52; V:43 | Preoperative | MRI | QDA | AUC=0.73 |
| 10.1002/jmri.27140 | Response | Retrospective | T:60 V:44 | Preoperative | MRI | RF | AUC=0.71 |
| 10.1038/s41598-019-46286-6 | Prognosis | Retrospective | 64 | Preoperative | CT | Cox | —— |
| 10.1038/s41598-018-30657-6 | Response | Retrospective | 95 | Preoperative | CT | DNN | AUC=0.72 |
| 10.1007/s11547-018-0951-y | Response | Retrospective | 16 | Longitudinal | MRI | —— | —— |
| 10.1016/j.radonc.2019.07.033 | Response | Prospective | T:70 V:55 | Preoperative | MRI | LR | AUC=0.86 |
| 10.3389/fmolb.2020.613918 | Prognosis | Retrospective | T:242 V:139 | Preoperative | CT | Cox | C-index=0.82 |
| 10.1007/s00330-020-07403-6 | Prognosis | Retrospective | T:119 V:30 | Preoperative | MRI | LR | AUC=0.79 |
| 10.1007/s11307-018-1225-8 | Prognosis | Retrospective | 74 | Preoperative | PET-CT | LR | AUC=0.71 |
| 10.1016/j.lfs.2018.07.007 | Prognosis | Prospective | T:81 V:34 | Preoperative | ERUS, CT | LR | C-index=0.86 |
| 10.1007/s00330-019-06558-1 | Prognosis | Prospective | T:29 V:11 | Preoperative | ERUS, MRI | ANN | Accuracy=0.75 |
| 10.3389/fonc.2020.575422 | Prognosis | Retrospective | T:99 V:42 | Preoperative | CT | LR | AUC=0.77 |
| 10.1007/s00330-018-5683-9 | Response | Retrospective | T:131 V:55 | Preoperative | MRI | LR | AUC=0.97 |
| 10.1007/s00330-019-06572-3 | Prognosis | Retrospective | T:213 V:177 | Preoperative | MRI | SVM | AUC=0.71 |
| 10.1007/s11547-017-0838-3 | Response | Retrospective | T:173 V:25 | Preoperative | MRI | LR | AUC=0.79 |
| 10.1002/ctm2.31 | Prognosis | Retrospective | 701 | Preoperative | CT | Cox | AUC=0.77, 0.744 |
| 10.1007/s00330-020-07673-0 | Prognosis | Retrospective | T:44 V:11 | Preoperative | MRI | RF | AUC=0.94 |
| 10.1093/jnC-index/djaa017 | Response | Prospective | T:445 V:222 | Preoperative , Postoperative | CT | RF | AUC=0.83 |
| 10.1016/j.ijrobp.2018.04.065 | Response | Retrospective | T:162 V:59 | Preoperative | MRI | LR, Cox | AUC=0.75 |
| 10.1136/gutjnl-2018-316407 | Response | Retrospective | T:120 V:110 | Preoperative , Postoperative | CT | Cox | C-index=0·75 |
| 10.21037/tcr.2019.11.41 | Staging | Retrospective | 26 | Preoperative | CT, MRI | LR | AUC=0.85 |
| 10.1186/s40644-020-00308-z | Prognosis | Retrospective | T:312 V:78 | Preoperative | CT | SVM | Accuracy=0.79 |
| 10.1016/j.acra.2019.02.009 | Prognosis | Retrospective | 119 | Preoperative | CT | Bayes | AUC=0.75 |
| 10.1088/1361-6560/ab7970 | Response | Retrospective | 43 | Preoperative | MRI | LR | AUC=0.73 |
| 10.1109/EMBC44109.2020.9176627 | Response | Retrospective | 95 | Preoperative | CT | SVM | Sensitivity=0.73 |
| 10.1007/s00259-018-4250-6 | Response | Retrospective | 52 | Preoperative | MRI,PET | LR | AUC=0.86 |
| 10.1007/s00261-019-02117-w | Prognosis | Retrospective | T:139 V:59 | Preoperative | CT | RF | AUC=0.79 |
| 10.1148/radiol.2021202363 | Diagnosis | Retrospective | T:63 V:59 | Preoperative | CT | RF | AUC=0.91 |
| 10.1186/s12880-020-00457-4 | Prognosis | Retrospective | T:117 V:40 | Preoperative | CT | ResNet | AUC=0.82 |
| 10.1002/jcp.28650 | Staging | Retrospective | T:88 V:30 | Preoperative | MRI | RF | AUC=0.72, 0.83 |
| 10.1016/j.ejrad.2019.02.022 | Prognosis | Retrospective | 65 | Preoperative | MRI | K-means | —— |
| 10.1148/radiol.2018172300 | Response | Retrospective | 114 | Preoperative | MRI | RF | AUC=0.93 |
| 10.1007/s00330-018-5539-3 | Prognosis | Retrospective | T:136 V:58 | Preoperative | CT | LR | AUC=0.92 |
| 10.1200/JCO.2015.65.9128 | Prognosis | Retrospective | T:326 V:200 | Preoperative | CT | LR | C-index=0.78 |
| 10.21147/j.issn.1000-9604.2018.01.05 | Prognosis | Retrospective | T:346 V:217 | Preoperative | CT | LR | C-index=0.80 |
| 10.1016/j.acra.2018.01.020 | Staging | Retrospective | T:222 V:144 | Preoperative | CT | LR | AUC=0.74 |
| 10.3390/cancers13030392 | Prognosis | Retrospective | T:228 V:153 | Preoperative | PET | Cox | C-index=0.72 |
| 10.1016/j.ejrad.2020.109326 | Prognosis | Retrospective | T:585 V:333 | Preoperative | CT | LR | AUC=0.78 |
| 10.21037/qims.2019.12.16 | Prognosis | Retrospective | T:80 V:20 | Preoperative | CT | LR | AUC=0.86 |
| 10.1002/cam4.2636 | Response | Retrospective | T:87 V：44 | Preoperative , Postoperative | MRI | LR | AUC=0.87 |
| 10.1007/s00432-020-03354-z | Prognosis | Retrospective | T:166 V:41 | Preoperative | CT | SVM | AUC=0.86 |
| 10.1186/s12967-020-02215-0 | Prognosis | Retrospective | T:458 V:308 | Preoperative | CT | LR | AUC=0.76 |
| 10.18632/oncotarget.8919 | Staging | Retrospective | T:286 V:206 | Preoperative | CT | LR | AUC=0.71 |
| 10.1016/j.acra.2018.12.019 | Prognosis | Retrospective | 108 | Preoperative | MRI | LR | AUC=0.87 |
| 10.1097/MD.0000000000019251 | Prognosis | Retrospective | 15 | Preoperative | CT | LR | AUC=0.88 |
| 10.1007/s00330-018-5802-7 | Prognosis | Retrospective | T:123 V:54 | Preoperative | CT, MRI | LR | AUC=0.83 |
| 10.1038/s41467-020-18162-9 | Prognosis | Retrospective | T:176 V:453 | Preoperative | MRI | Cox | C-index=0.85, 0.83 |
| 10.1158/1078-0432.CCR-17-1038 | Response | Retrospective | T:152 V:70 | Preoperative , Postoperative | MRI | LR | AUC=0.98 |
| 10.1007/s00259-017-3855-5 | Prognosis | Retrospective | 86 | Preoperative | PET-CT | —— | —— |
| 10.1186/s12880-019-0392-7 | Staging | Retrospective | T:106 V：46 | Preoperative | MRI | SVM | AUC=0.86 |
| 10.1007/s00330-018-5763-x | Prognosis | Retrospective | T:197 V:148 | Preoperative | MRI | LR | AUC=0.70 |
| 10.1245/s10434-020-08974-w | Prognosis | Retrospective | T:175 V:72 | Preoperative | CT | LR | AUC=0.90 |
| 10.1158/1078-0432.CCR-15-2997 | Response | Retrospective | 48 | Preoperative | MRI | ANN | AUC=0.84 |
| 10.3390/cancers12102978 | Response | Retrospective | T:36 V:9 | Preoperative , Intraoperative | PET,CT | RF | AUC=0.80 |
| 10.4143/crt.2019.050 | Prognosis | Retrospective | 60 | Preoperative | MR | DT | Accuracy=0.81 |
| 10.1007/s00261-020-02502-w | Response | Retrospective | 102 | Preoperative | MRI | SVM | Accuracy=0.74 |
| 10.1016/j.ejmp.2019.03.013 | Response | Retrospective | T:53 V:45 | Preoperative | MRI | Ensemble (SVM, BN, NN, KNN) | AUC=0.95 |
| PMID: 33415015 | Prognosis | Retrospective | T:124 V:35 | Preoperative | CT | ANN | AUC=0.79 |
| 10.1016/j.mri.2019.05.003 | Response | Retrospective | 86 | Preoperative , Postoperative | MRI | CNN | AUC=0.83 |
| 10.1038/s41598-019-39651-y | Prognosis | Retrospective | T:135 V:159 | Preoperative | MRI | LR | AUC=0.91 |
| 10.1016/j.radonc.2019.03.011 | Response | Retrospective | T:79 V:42 | Preoperative | CT | LR | AUC=0.70 |
| 10.1038/s41598-019-51629-4 | Response | Retrospective | 411 | Preoperative | CT | NMF | C-index=0.73 |
| 10.3389/fonc.2019.01250 | Prognosis | Retrospective | T:69 V:33 | Preoperative | CT | LR | AUC=0.92 |
| 10.1016/j.acra.2019.12.007 | Prognosis | Retrospective | T:276 V:119 | Preoperative | CT | LR | C-index=0.83 |
| 10.1007/s00384-019-03455-3 | Response | Retrospective | T:66 V:23 | Preoperative | MRI | RF | AUC=0.83 |
| 10.1007/s00330-017-5146-8 | Prognosis | Retrospective | T:61 V:56 | Preoperative | CT | SVM | AUC=0.83 |
| 10.1007/s11307-020-01552-2 | Response | Retrospective | T:56 V:24 | Preoperative | CT | Cox | C-index=0.92 |
| 10.1245/s10434-019-07300-3 | Response | Retrospective | T:318 V:107 | Preoperative | MRI | LR | AUC=0.82 |
| 10.3389/fonc.2021.687771 | Response | Retrospective | T:441;V:61 | Preoperative | CT | LR | AUC=0.97 |
| 10.1038/s41598-022-10175-2 | Response | Retrospective | 191 | Preoperative | CT | Cox | AUC=0.68 |
| 10.21037/atm.2020.01.107 | Response | Retrospective | 169 | Preoperative | PET-CT | RF | Accuracy=0.95 |
| 10.1016/j.radonc.2019.07.033 | Response | Prospective | T:70;V:55 | Preoperative | MRI | LR | AUC=0.86 |
| 10.3389/fonc.2021.671636 | Response | Retrospective | T:52;V:40 | Preoperative | MRI | LR | AUC=0.87 |
| 10.1007/s00330-021-08104-4 | Prognosis | Retrospective | T:136;V:57 | Preoperative | CT | LR | AUC=0.78 |
| 10.1007/s12029-019-00291-0 | Response | Retrospective | 67 | Preoperative | MRI | BN | AUC=0.85 |
| 10.1007/s00261-021-03021-y | Prognosis | Retrospective | T:65;V:29 | Preoperative | MRI | LR | AUC=0.90 |
| 10.1016/j.acra.2021.12.016 | Prognosis | Retrospective | T:98;V:42 | Preoperative | CT | LR | AUC=0.88 |
| 10.3389/fonc.2020.00459 | Prognosis | Retrospective | T:74;V:32 | Preoperative | MRI | LR | AUC=0.81 |
| 10.1259/bjr.20211014 | Prognosis | Retrospective | T:121;V:51 | Preoperative | CT | LR | AUC=0.92 |
| 10.3389/fonc.2022.828904 | Prognosis | Retrospective | T:194;V:85 | Preoperative | MRI | LR | AUC=0.86 |
| 10.1038/s41598-021-88831-2 | Prognosis | Retrospective | T:65;V:29 | Preoperative | CT, MRI | LR | AUC=0.88 |
| 10.1007/s12149-021-01605-8 | Prognosis | Retrospective | T:144;V:55 | Preoperative | PET-CT | LR | AUC=0.73 |
| 10.5603/RPOR.a2021.0004 | Response | Retrospective | 91 | Preoperative | CT, PET | LR | AUC=0.95, 0.81 |
| 10.1007/s00261-020-02710-4 | Prognosis | Retrospective | T:87;V:35 | Preoperative | MRI | LR | AUC=0.85 |
| 10.1002/jmri.26972 | Prognosis | Retrospective | T:85;V:37 | Preoperative | MRI | LR | AUC=0.86 |
| 10.1007/s12149-021-01622-7 | Prognosis | Retrospective | 94 | Preoperative | PET-CT | —— | —— |
| 10.3233/XST-210877 | Diagnosis | Retrospective | T:110;V:59 | Preoperative | CT | LR | AUC=0.90 |
| 10.1016/j.radonc.2020.09.039 | Response | Retrospective | T:131;V:53 | Preoperative | MRI | Cox | C-index=0.80 |
| 10.3389/fonc.2021.702055 | Prognosis | Retrospective | 173 | Preoperative | PET-CT | Adboost | AUC=0.83 |
| 10.1186/s12967-022-03262-5 | Prognosis | Retrospective | T:137;V:59 | Preoperative | PET-CT | RF | C-index=0.82 |
| 10.3389/fonc.2020.00457 | Prognosis | Retrospective | T:65;V:29 | Preoperative | CT, MRI | LR | AUC=0.88 |
| 10.1002/jmri.25969 | Prognosis | Retrospective | T:73;V:24 | Preoperative | MRI | LR | AUC=0.85 |
| 10.1186/s12967-021-02919-x | Response | Prospective | T:113;V:64 | Preoperative | CT, MRI | GBM | AUC=0.83 |
| 10.1007/S00261-022-03477-6 | Staging | Retrospective | T:60;V:144 | Preoperative | MRI | XGBoost | AUC=0.92 |
| 10.1186/S12880-022-00773-X | Response | Retrospective | 88 | Preoperative | MRI | GLM | AUC=0.93 |
| 10.1186/S12885-022-09518-Z | Response | Retrospective | T:129;V:78 | Preoperative | MRI | SVM | AUC=0.92 |
| 10.3389/FONC.2021.610338 | Prognosis | Retrospective | T:198;V:83 | Preoperative | CT, MRI | LR | AUC=0.83 |
| 10.1007/S00330-021-08144-W | Prognosis | Retrospective | 236 | Preoperative | MRI | SVM | AUC=0.89 |
| 10.1016/j.adro.2020.04.016 | Response | Retrospective | T:26;V:13 | Preoperative | MRI | SVM | —— |
| 10.1038/S41416-022-01786-7 | Response | Retrospective | 735 | Preoperative | MRI | SVM | AUC=0.79 |
| 10.1148/RADIOL.211986 | Response | Retrospective | T:592;V:306 | Preoperative | MRI | LR | AUC=0.82 |
| 10.3389/FONC.2021.614052 | Prognosis | Retrospective | T:58;V:25 | Preoperative | MRI | LR | C-index=0.70 |
| 10.1038/S41598-021-84816-3 | Response | Retrospective | 72 | Preoperative | MRI | PLS | AUC=0.79 |
| 10.1016/j.acra.2020.10.026 | Response | Retrospective | T:116;V:49 | Longitudinal | MRI | LR | AUC=0.91 |
| 10.3389/FONC.2022.801743 | Response | Prospective | T:91;V:46 | Preoperative | MRI | LR | AUC=0.84 |
| 10.1016/j.acra.2020.09.024 | Prognosis | Retrospective | T:40;V:40 | Preoperative | MRI | XGBoost | AUC=0.86 |
| 10.3389/fonc.2019.00552 | Response | Retrospective | T:70;V:48 | Preoperative | MRI | SVM | AUC=0.87 |
| 10.3748/wjg.v26.i19.2388 | Response | Retrospective | T:70;V:48 | Preoperative | CT, MRI | LR | AUC=0.93 |
| 10.1007/S00261-021-03397-X | Prognosis | Prospective | 110 | Preoperative | MRI | SVM | C-index=0.70 |
| 10.3389/FGENE.2022.880093 | Prognosis | Retrospective | T:426;V:382 | Preoperative | CT | MSCNN | AUC=0.61 |
| 10.1007/S00261-021-03219-0 | Response | Retrospective | T:129;V:64 | Preoperative | MRI | LR | AUC=0.94 |
| 10.1007/s00330-020-07403-6 | Prognosis | Retrospective | T:119;V:30 | Preoperative | MRI | LR | AUC=0.79 |
| 10.3389/FONC.2020.585767 | Prognosis | Retrospective | T:123;V:63 | Preoperative | MRI | SVM | AUC=0.83 |
| 10.1007/S00330-022-08954-6 | Prognosis | Retrospective | T:560;V:277 | Preoperative | CT | LR | AUC=0.78,0.77 |
| 10.1002/jmri.25968 | Prognosis | Retrospective | T:54;V:54 | Preoperative | MRI | Cox | C-index=0.79 |
| 10.3389/FONC.2022.850774 | Response | Retrospective | T:151;V:65 | Preoperative | CT | LR | AUC=0.87 |
| 10.1007/S00330-021-08167-3 | Prognosis | Retrospective | T:534;V:228 | Preoperative | CT | LR | AUC=0.77 |
| 10.3390/cancers12071894 | Response | Retrospective | 67 | Preoperative | MRI | LR | AUC=0.80 |
| 10.1016/j.ejrad.2020.109205 | Prognosis | Retrospective | T:322;V:250 | Preoperative | CT | Cox | AUC=0.68 |
| 10.1007/s00330-021-07877-y | Diagnosis | Retrospective | T:386;V:116 | Preoperative | CT | LR | AUC=0.94 |
| 10.3389/fonc.2021.697497 | Prognosis | Prospective | T:322;V:250 | Preoperative | MRI | LR | AUC=0.93 |
| 10.3233/XST-190614 | Diagnosis | Retrospective | T:155;V:70 | Preoperative | CT | LR | AUC=0.93 |
| 10.3389/fonc.2021.671354 | Prognosis | Retrospective | T:97;V:65 | Preoperative | MRI | SVM、LR | AUC=0.88 |
| 10.1016/j.acra.2021.11.024 | Prognosis | Retrospective | T:164;V:71 | Preoperative | MRI | Cox | C-index=0.78 |
| 10.3748/wjg.v27.i33.5610 | Prognosis | Retrospective | T:242;V:61 | Preoperative | CT | LR | AUC=0.80 |
| 10.3390/cancers11111680 | Response | Retrospective | 169 | Preoperative | CT | RF | —— |
| 10.1093/bjs/znab191 | Prognosis | Retrospective | T:98;V:48 | Preoperative | MRI | RF | AUC=0.69 |
| 10.1007/s00330-020-06968-6 | Response | Prospective | T:112;V:20 | Preoperative | MRI | LR | AUC=0.80 |
| 10.3748/wjg.v26.i33.5008 | Prognosis | Retrospective | T:103;V:45 | Preoperative | CT | LR | AUC=0.80 |
| 10.5152/dir.2021.19677 | Response | Retrospective | T:134;V:55 | Preoperative | MRI | RF | AUC=0.95 |
| PMC-indexD: PMC6682712 | Staging | Retrospective | T:136;V:59 | Preoperative | CT | SVM | AUC=0.75 |
| 10.1148/rycan.2020190084 | Prognosis | Retrospective | 145 | Preoperative | CT | —— | —— |
| 10.3389/fonc.2020.00604 | Prognosis | Retrospective | T:261;V:130 | Preoperative | MRI | LR | AUC=0.82 |
| 10.1158/1078-0432.CCR-15-2997 | Response | Retrospective | 48 | Preoperative | MRI | ANN | AUC=0.89 |
| 10.1002/jmri.28108 | Response | Retrospective | T:107;V:46 | Preoperative | MRI | LR | AUC=0.90 |
| 10.1186/s13014-022-02053-y | Response | Retrospective | T:126;V:75 | Preoperative | CT | RF | AUC=0.63 |
| 10.1007/s00261-021-03311-5 | Prognosis | Retrospective | T:77;V:51 | Preoperative | MRI | SVM | AUC=0.88 |
| 10.1007/s00330-020-07192-y | Prognosis | Retrospective | 103 | Preoperative | CT | GMS | AUC=0.73 |
| 10.1007/s00261-022-03453-0 | Prognosis | Retrospective | T:92;V:39 | Preoperative | PET-CT | LR、Cox | AUC=0.80 |
| 10.1002/mp.14896 | Response | Retrospective | T:36;V:17 | Longitudinal | MRI | RF | AUC=0.96 |
| 10.18632/oncotarget.13855 | Prognosis | Retrospective | 122 | Preoperative | MRI | LR | AUC=0.80 |
| 10.1002/MP.15001 | Prognosis | Retrospective | T:149;V:64 | Preoperative | MRI | LR | AUC=0.88, 0.83 |
| 10.1016/j.acra.2022.02.00 | Prognosis | Retrospective | T:121;V:165 | Preoperative | CT | Cox | C-index=0.72, 0.68 |
| 10.3389/fonc.2020.574337 | Response | Retrospective | T:500;V:200 | Preoperative | MRI | CNN | AUC=0.85 |
| 10.3389/fonc.2020.00457 | Prognosis | Retrospective | T:65;V:29 | Preoperative | CT, MRI | LR | AUC=0.88 |
| 10.3389/fonc.2022.831712 | Prognosis | Retrospective | 48 | Longitudinal | MR | LR | AUC=0.93 |
| 10.1016/J.RADONC.2021.07.004 | Prognosis | Retrospective | T:75;V:38 | Preoperative | MRI | LR | AUC=0.73, 0.82 |
| 10.1007/s00261-021-03137-1 | Staging | Prospective | T:188;V:80 | Preoperative | MRI | LR | AUC=0.85, 0.86 |
| 10.3390/cancers14123004 | Response | Retrospective | T:37;V:63 | Longitudinal | MRI | LR | AUC=0.92, 0.88 |
| 10.3390/cancers14081895 | Prognosis | Retrospective | 71 | Preoperative | CT | CATC | AUC=0.56 |
| 10.1186/S12880-022-00813-6 | Prognosis | Retrospective | T:143;V:60 | Preoperative | ERUS | LR | AUC=0.78 |
| 10.1007/S00261-020-02863-2 | Prognosis | Retrospective | 91 | Preoperative | MRI | LR | AUC=0.94 |
| 10.1186/S13014-022-02048-9 | Response | Retrospective | T:161;V:59 | Preoperative | MRI | LR | AUC=0.82, 0.86 |
| 10.3389/FONC.2022.861892 | Prognosis | Retrospective | T:171;V:142 | Preoperative | CT | MPC | AUC=0.79, 0.72 |
| 10.3389/FONC.2022.843991 | Response | Retrospective | T:97;V:42 | Longitudinal | CT | Cox | AUC=0.74 |
| 10.3390/cancers14092231 | Response | Retrospective | T:30;V:13 | Preoperative | MRI | SVM | AUC=0.89 |
| 10.4254/wjh.v14.i1.244 | Response | Retrospective | T:15;V:14 | Preoperative | CT | LR | AUC=0.77 |
| 10.3389/FONC.2021.694102 | Response | Retrospective | T:56;V:24 | Preoperative | US | RF | AUC=0.94 |
| 10.1007/S11307-022-01730-4 | Prognosis | Retrospective | T:127;V:64 | Preoperative | CT | LR | AUC=0.71 |
| 10.1007/s00261-022-03572-8 | Response | Retrospective | T:292;V:82 | Preoperative | MRI | RF | AUC=0.79 |
| 10.21873/invivo.12126 | Response | Retrospective | T:90;V:45 | Preoperative | MRI | EN | AUC=0.71 |
| 10.1016/J.TRANON.2022.101352 | Prognosis | Retrospective | T:146;V:60 | Preoperative | MRI | Cox | C-index=0.94 |
| 10.1016/J.EJRAD.2021.110065 | Prognosis | Retrospective | T:239;V:112 | Preoperative | CT | LR | AUC=0.84, 0.81 |
| 10.3389/FONC.2021.710248 | Prognosis | Retrospective | T:203;V:51 | Preoperative | CT | LR | AUC=0.83 |
| 10.3389/fonc.2021.666786 | Prognosis | Retrospective | T:226;V:142 | Preoperative | CT | LR | AUC=0.73 |
| 10.3390/cancers14051110 | Response | Retrospective | T:51;V:30 | Preoperative | MRI | KNN、DT | AUC=0.97 |
| 10.21147/j.issn.1000-9604.2022.01.04 | Prognosis | Retrospective | T:161;V:248 | Preoperative | CT | Cox | C-index=0.76, 0.75 |
| 10.1111/1754-9485.13044 | Response | Retrospective | T:60;V:31 | Preoperative | CT | RF | Accuracy=0.84 |
| 10.3389/fonc.2021.644933 | Prognosis | Retrospective | T:210;V:89 | Preoperative | CT | Cox | AUC=0.91 |
| 10.1016/j.ebiom.2021.103442 | Prognosis | Retrospective | T:170;V:65 | Preoperative | MRI | ResNet | C-index=0.78 |
| 10.1007/s00330-022-08952-8 | Staging | Retrospective | T:565;V:141 | Preoperative | MRI | LR | AUC=0.87 |
| 10.3389/fonc.2020.595012 | Prognosis | Retrospective | T:192;V:21 | Longitudinal | MRI | LR | AUC=0.79 |
| 10.1016/j.ejmp.2021.03.038 | Response | Retrospective | T:16;V:43 | Longitudinal | MRI | —— | AUC=0.93 |
| 10.1186/s13014-019-1246-8 | Response | Retrospective | T:67;V:34 | Longitudinal | MRI | LR | AUC=0.93 |
| 10.1186/s12885-022-09584-3 | Prognosis | Retrospective | T:194;V:82 | Preoperative | CT | LR | AUC=0.90 |
| 10.1007/s00261-020-02846-3 | Response | Retrospective | T:122;V:61 | Preoperative | MRI | LR | AUC=0.90 |
| 10.21037/atm-20-7673 | Prognosis | Retrospective | T:327;V:64 | Preoperative | MRI | XGBoost | AUC=0.90 |
| 10.2147/CMAR.S295317 | Response | Retrospective | T:107;V:82 | Preoperative | MRI | SVM | AUC=0.91 |
| 10.1007/s11547-021-01421-0 | Response | Retrospective | 144 | Preoperative | MRI | LR | AUC=0.84 |
| 10.1186/s12880-021-00560-0 | Response | Retrospective | 114 | Preoperative | MRI | RF、KNN | AUC=0.95 |
| 10.1155/2021/4520450 | Prognosis | Retrospective | 98 | Preoperative | MRI | CNN | AUC=0.76 |
| 10.3390/cancers14041079 | Response | Retrospective | T:64;V:60 | Preoperative | CT, MRI | CNN | AUC=0.95 |
| 10.1007/s11596-020-2298-6 | Prognosis | Retrospective | T:83;V:44 | Preoperative | MRI | LDA | AUC=0.67 |
| 10.3389/fonc.2021.774413 | Response | Retrospective | T:125;V:63 | Preoperative | MRI | LR | AUC=0.75 |
| 10.1186/s40644-021-00408-4 | Prognosis | Retrospective | T:99;V:41 | Preoperative | MRI | LR | AUC=0.75 |
| 10.1007/s00261-020-02733-x | Prognosis | Retrospective | T:98;V:41 | Preoperative | MRI | LR | AUC=0.87 |
| 10.1007/s00261-022-03534-0 | Prognosis | Retrospective | 292 | Preoperative | CT | LR | AUC=0.73 |
| 10.3389/fonc.2021.620945 | Prognosis | Retrospective | T:62;V:72 | Preoperative | CT | LR | AUC=0.82 |
| 10.3390/cancers12082027 | Response | Retrospective | T:52;V:43 | Preoperative | MRI | QDA | AUC=0.73 |

**Abbreviations:** ANN = artificial neural network, AUC = area under the receiver operator characteristic curve, BN=Bayesian network , BNN = Bayesian neural network, CATC= covariate-adjusted tensor classification, C-index=concordance index, CNN = convolutional neural network, COX=proportional hazards model, CT= computed tomography, DNN=dynamic neural network, DT= decision tree, ERUS= endorectal ultrasonography, EN= elastic net, GBM= gradient boosting machine, GLM=generalized linear model, HR= hazard ratio, KNN = k-nearest neighbor, LDA= linear discriminant Analysis, LNMF=local nonnegative matrix factorization, LR = logistic regression model, MRI=magnetic resonance imaging, MPC= multilayer perceptron classifier, MSCNN= Multisize convolutional neural network, NMF=nonnegative matrix factorization, NN=neural network, PET-CT=positron emission tomography/ computed tomography, PLS= Partial least square regression, QDA= quadratic discriminant analysis, RF = random forest, ResNet =residual networks, SVM = supporter vector machine, SWE=shear wave elastography, T= training set, V= verification set.

**Additional Table 2: RQS of studies**

| Reference | Image protocol | Multiple segmentations | Phantom study | Multiple time points | Feature reduction | Multivariable analysis | Biological correlates | Cut-off analysis | Discriminative statistics | Calibration statistics | Prospective study | Validation | Gold standard Comparision | Clinical utility | Cost-effectiveness | Open science data | Total score |
| --- | --- | --- | --- | --- | --- | --- | --- | --- | --- | --- | --- | --- | --- | --- | --- | --- | --- |
| 10.1016/j.ejrad.2019.02.022 | 1 | 1 | 0 | 0 | 3 | 0 | 1 | 0 | 0 | 0 | 0 | -5 | 0 | 0 | 0 | 1 | 2 |
| 10.1148/radiol.2018172300 | 1 | 1 | 0 | 0 | 3 | 0 | 0 | 0 | 2 | 0 | 0 | -5 | 0 | 0 | 0 | 1 | 3 |
| 10.1007/s00330-018-5539-3 | 1 | 1 | 0 | 0 | 3 | 1 | 0 | 0 | 2 | 1 | 0 | 2 | 0 | 0 | 0 | 1 | 12 |
| 10.1200/JCO.2015.65.9128 | 1 | 1 | 0 | 0 | 3 | 1 | 0 | 0 | 2 | 1 | 0 | 2 | 0 | 2 | 0 | 1 | 14 |
| 10.21147/j.issn.1000-9604.2018.01.05 | 1 | 1 | 0 | 0 | 3 | 1 | 0 | 0 | 2 | 1 | 0 | 2 | 0 | 2 | 0 | 1 | 14 |
| 10.1016/j.acra.2018.01.020 | 1 | 1 | 0 | 0 | 3 | 0 | 0 | 1 | 2 | 0 | 0 | 2 | 0 | 0 | 0 | 1 | 11 |
| 10.3390/cancers13030392 | 1 | 1 | 1 | 0 | 3 | 1 | 0 | 0 | 2 | 0 | 0 | 2 | 0 | 2 | 0 | 1 | 14 |
| 10.1016/j.ejrad.2020.109326 | 1 | 1 | 0 | 0 | 3 | 1 | 0 | 0 | 1 | 1 | 0 | 2 | 0 | 2 | 0 | 1 | 13 |
| 10.21037/qims.2019.12.16 | 1 | 1 | 0 | 0 | 3 | 1 | 0 | 0 | 2 | 0 | 0 | 2 | 0 | 2 | 0 | 1 | 13 |
| 10.1002/cam4.2636 | 1 | 1 | 0 | 0 | 3 | 0 | 0 | 0 | 2 | 2 | 0 | 2 | 0 | 0 | 0 | 2 | 13 |
| 10.1007/s00432-020-03354-z | 1 | 1 | 0 | 0 | 3 | 0 | 1 | 0 | 2 | 0 | 0 | 2 | 0 | 2 | 0 | 1 | 13 |
| 10.1186/s12967-020-02215-0 | 1 | 1 | 0 | 0 | 3 | 1 | 0 | 0 | 2 | 0 | 0 | 2 | 0 | 2 | 0 | 1 | 13 |
| 10.18632/oncotarget.8919 | 1 | 0 | 0 | 0 | 3 | 1 | 0 | 0 | 2 | 0 | 0 | 2 | 0 | 0 | 0 | 1 | 10 |
| 10.1016/j.acra.2018.12.019 | 1 | 1 | 0 | 0 | 3 | 0 | 0 | 0 | 2 | 0 | 0 | -5 | 0 | 0 | 0 | 1 | 3 |
| 10.1097/MD.0000000000019251 | 1 | 0 | 0 | 0 | 3 | 0 | 1 | 0 | 2 | 0 | 0 | -5 | 0 | 0 | 0 | 1 | 3 |
| 10.1007/s00330-018-5802-7 | 1 | 1 | 0 | 0 | 3 | 1 | 0 | 0 | 2 | 0 | 0 | 2 | 0 | 2 | 0 | 1 | 13 |
| 10.1038/s41467-020-18162-9 | 1 | 1 | 0 | 0 | 3 | 1 | 0 | 1 | 2 | 1 | 0 | 5 | 0 | 2 | 0 | 2 | 19 |
| 10.1158/1078-0432.CCR-17-1038 | 1 | 1 | 0 | 0 | 3 | 1 | 0 | 0 | 2 | 2 | 0 | 2 | 0 | 2 | 0 | 1 | 15 |
| 10.1007/s00259-017-3855-5 | 2 | 0 | 0 | 0 | 3 | 1 | 1 | 0 | 0 | 0 | 0 | -5 | 0 | 0 | 0 | 1 | 3 |
| 10.1186/s12880-019-0392-7 | 1 | 0 | 0 | 1 | 3 | 0 | 0 | 0 | 2 | 0 | 0 | 2 | 0 | 0 | 0 | 1 | 10 |
| 10.1007/s00330-018-5763-x | 1 | 1 | 0 | 0 | 3 | 1 | 1 | 0 | 2 | 0 | 0 | 2 | 0 | 0 | 0 | 2 | 13 |
| 10.1245/s10434-020-08974-w | 1 | 1 | 0 | 0 | 3 | 1 | 0 | 0 | 1 | 2 | 0 | 3 | 2 | 0 | 0 | 1 | 15 |
| 10.1158/1078-0432.CCR-15-2997 | 1 | 0 | 0 | 0 | 3 | 0 | 0 | 0 | 1 | 0 | 0 | -5 | 0 | 0 | 0 | 1 | 1 |
| 10.3390/cancers12102978 | 1 | 1 | 0 | 0 | 3 | 0 | 1 | 0 | 2 | 0 | 0 | 2 | 0 | 0 | 0 | 1 | 11 |
| 10.4143/crt.2019.050 | 1 | 1 | 0 | 0 | 3 | 0 | 1 | 0 | 1 | 0 | 0 | -5 | 0 | 0 | 0 | 1 | 3 |
| 10.1007/s00261-020-02502-w | 1 | 1 | 0 | 0 | 3 | 0 | 0 | 0 | 1 | 0 | 0 | -5 | 0 | 2 | 0 | 2 | 5 |
| 10.1016/j.ejmp.2019.03.013 | 1 | 1 | 0 | 0 | 3 | 0 | 0 | 0 | 1 | 0 | 0 | 2 | 0 | 0 | 0 | 1 | 9 |
| PMID: 33415015 | 1 | 1 | 0 | 0 | 3 | 0 | 1 | 0 | 1 | 1 | 0 | 2 | 0 | 0 | 0 | 1 | 11 |
| 10.1016/j.mri.2019.05.003 | 1 | 0 | 0 | 0 | 3 | 0 | 0 | 0 | 2 | 0 | 0 | -5 | 0 | 0 | 0 | 1 | 2 |
| 10.1038/s41598-019-39651-y | 1 | 1 | 0 | 0 | 3 | 1 | 0 | 0 | 2 | 1 | 0 | 2 | 0 | 2 | 0 | 1 | 14 |
| 10.1016/j.radonc.2019.03.011 | 0 | 0 | 0 | 0 | 3 | 1 | 0 | 0 | 2 | 1 | 0 | 2 | 0 | 0 | 0 | 0 | 9 |
| 10.1038/s41598-019-51629-4 | 0 | 1 | 0 | 1 | 3 | 1 | 0 | 0 | 2 | 0 | 0 | -5 | 0 | 2 | 0 | 0 | 5 |
| 10.3389/fonc.2019.01250 | 1 | 1 | 0 | 0 | 3 | 0 | 1 | 0 | 1 | 1 | 0 | 2 | 0 | 2 | 0 | 1 | 13 |
| 10.1016/j.acra.2019.12.007 | 1 | 1 | 0 | 0 | 3 | 1 | 1 | 0 | 2 | 1 | 0 | 2 | 0 | 2 | 0 | 1 | 15 |
| 10.1007/s00384-019-03455-3 | 1 | 1 | 0 | 0 | 3 | 1 | 0 | 0 | 1 | 0 | 0 | 2 | 0 | 0 | 0 | 1 | 10 |
| 10.1007/s00330-017-5146-8 | 1 | 1 | 0 | 0 | 3 | 1 | 0 | 0 | 2 | 0 | 0 | 2 | 0 | 0 | 0 | 1 | 11 |
| 10.1007/s11307-020-01552-2 | 1 | 0 | 0 | 0 | 3 | 1 | 0 | 1 | 2 | 1 | 0 | 2 | 0 | 2 | 0 | 1 | 14 |
| 10.1245/s10434-019-07300-3 | 1 | 1 | 0 | 0 | 3 | 1 | 0 | 0 | 2 | 1 | 0 | 2 | 0 | 0 | 0 | 1 | 12 |
| 10.3389/fonc.2021.687771 | 1 | 1 | 0 | 0 | 3 | 1 | 1 | 0 | 1 | 1 | 0 | 3 | 0 | 2 | 0 | 1 | 15 |
| 10.1038/s41598-022-10175-2 | 1 | 0 | 0 | 0 | 3 | 1 | 1 | 0 | 2 | 0 | 0 | -5 | 0 | 0 | 0 | 1 | 4 |
| 10.21037/atm.2020.01.107 | 1 | 1 | 0 | 0 | 3 | 0 | 0 | 0 | 2 | 0 | 0 | -5 | 0 | 0 | 0 | 1 | 3 |
| 10.1016/j.radonc.2019.07.033 | 1 | 1 | 0 | 0 | 3 | 0 | 0 | 0 | 2 | 0 | 7 | 2 | 0 | 0 | 0 | 1 | 17 |
| 10.3389/fonc.2021.671636 | 1 | 1 | 0 | 0 | 3 | 1 | 0 | 0 | 1 | 1 | 0 | 3 | 0 | 2 | 0 | 1 | 14 |
| 10.1007/s00330-021-08104-4 | 1 | 0 | 0 | 0 | 3 | 1 | 0 | 0 | 1 | 0 | 0 | 3 | 0 | 0 | 0 | 1 | 10 |
| 10.1007/s12029-019-00291-0 | 1 | 1 | 0 | 0 | 3 | 0 | 0 | 0 | 2 | 0 | 0 | -5 | 0 | 0 | 0 | 1 | 3 |
| 10.1007/s00261-021-03021-y | 1 | 1 | 0 | 0 | 3 | 1 | 0 | 0 | 1 | 1 | 0 | 2 | 0 | 2 | 0 | 1 | 13 |
| 10.1016/j.acra.2021.12.016 | 1 | 1 | 0 | 0 | 3 | 1 | 1 | 0 | 1 | 1 | 0 | 2 | 0 | 2 | 0 | 1 | 14 |
| 10.3389/fonc.2020.00459 | 1 | 0 | 0 | 0 | 3 | 1 | 1 | 0 | 1 | 1 | 0 | 2 | 0 | 2 | 0 | 1 | 13 |
| 10.1259/bjr.20211014 | 1 | 1 | 0 | 0 | 3 | 1 | 1 | 0 | 1 | 1 | 0 | 2 | 0 | 2 | 0 | 1 | 14 |
| 10.3389/fonc.2022.828904 | 1 | 1 | 0 | 0 | 3 | 1 | 1 | 0 | 1 | 1 | 0 | 2 | 0 | 2 | 0 | 1 | 14 |
| 10.1038/s41598-021-88831-2 | 1 | 1 | 0 | 0 | 3 | 0 | 1 | 0 | 1 | 1 | 0 | 2 | 0 | 2 | 0 | 1 | 13 |
| 10.1007/s12149-021-01605-8 | 1 | 1 | 0 | 0 | 3 | 1 | 0 | 0 | 1 | 1 | 0 | 2 | 0 | 0 | 0 | 1 | 11 |
| 10.5603/RPOR.a2021.0004 | 0 | 0 | 0 | 0 | 3 | 0 | 0 | 0 | 2 | 0 | 0 | -5 | 0 | 0 | 0 | 0 | 0 |
| 10.1007/s00261-020-02710-4 | 1 | 1 | 0 | 0 | 3 | 1 | 1 | 0 | 1 | 1 | 0 | 2 | 0 | 2 | 0 | 1 | 14 |
| 10.1002/jmri.26972 | 1 | 1 | 0 | 0 | 3 | 1 | 0 | 0 | 1 | 1 | 0 | 2 | 0 | 0 | 0 | 1 | 11 |
| 10.1007/s12149-021-01622-7 | 1 | 1 | 0 | 0 | 3 | 0 | 1 | 0 | 0 | 0 | 0 | -5 | 0 | 0 | 0 | 1 | 2 |
| 10.3233/XST-210877 | 1 | 1 | 0 | 0 | 3 | 1 | 0 | 0 | 1 | 1 | 0 | 3 | 0 | 2 | 0 | 1 | 14 |
| 10.1016/j.radonc.2020.09.039 | 1 | 0 | 0 | 0 | 3 | 1 | 1 | 0 | 1 | 1 | 0 | 2 | 0 | 2 | 0 | 1 | 13 |
| 10.3389/fonc.2021.702055 | 1 | 1 | 0 | 0 | 3 | 1 | 0 | 0 | 1 | 0 | 0 | -5 | 0 | 0 | 0 | 1 | 3 |
| 10.1186/s12967-022-03262-5 | 1 | 1 | 0 | 0 | 3 | 1 | 0 | 0 | 2 | 0 | 0 | 2 | 0 | 0 | 0 | 1 | 11 |
| 10.3389/fonc.2020.00457 | 1 | 1 | 0 | 0 | 3 | 0 | 0 | 0 | 1 | 1 | 0 | 2 | 0 | 2 | 0 | 1 | 12 |
| 10.1002/jmri.25969 | 1 | 1 | 0 | 0 | 3 | 0 | 0 | 0 | 1 | 0 | 0 | 2 | 0 | 0 | 0 | 1 | 9 |
| 10.1186/s12967-021-02919-x | 1 | 1 | 0 | 0 | 3 | 1 | 0 | 0 | 1 | 0 | 7 | 2 | 0 | 2 | 0 | 1 | 19 |
| 10.1007/S00261-022-03477-6 | 1 | 1 | 0 | 0 | 3 | 0 | 0 | 0 | 1 | 0 | 0 | 2 | 0 | 0 | 0 | 1 | 9 |
| 10.1186/S12880-022-00773-X | 1 | 0 | 0 | 0 | 3 | 0 | 0 | 0 | 2 | 0 | 0 | -5 | 0 | 0 | 0 | 1 | 2 |
| 10.1186/S12885-022-09518-Z | 1 | 1 | 0 | 0 | 3 | 0 | 0 | 0 | 1 | 0 | 0 | 2 | 0 | 0 | 0 | 2 | 10 |
| 10.3389/FONC.2021.610338 | 1 | 1 | 0 | 0 | 3 | 1 | 0 | 0 | 1 | 1 | 0 | 2 | 0 | 2 | 0 | 1 | 13 |
| 10.1007/S00330-021-08144-W | 1 | 1 | 0 | 0 | 3 | 0 | 0 | 0 | 2 | 0 | 0 | -5 | 0 | 0 | 0 | 1 | 3 |

**Additional Table 3: Risk of bias in individual studies**

| DOI/PMID | Method details provided | | | | | Code details provided | Repeatability | |
| --- | --- | --- | --- | --- | --- | --- | --- | --- |
|  | imaging | segmentation | features | methods | model performance |  | methods | external validation |
| 10.3390/cancers12082027 | Yes | Yes | Yes | Yes | Yes | No | No | Yes |
| 10.1002/jmri.27140 | Yes | Yes | Yes | Yes | Yes | No | No | Yes |
| 10.1038/s41598-019-46286-6 | Yes | No | Yes | Yes | No | No | No | No |
| 10.1038/s41598-018-30657-6 | Yes | Yes | Yes | Yes | Yes | No | No | No |
| 10.1007/s11547-018-0951-y | No | Yes | No | No | No | No | No | No |
| 10.1016/j.radonc.2019.07.033 | Yes | Yes | Yes | Yes | Yes | Yes | No | No |
| 10.3389/fmolb.2020.613918 | No | No | Yes | Yes | Yes | No | No | No |
| 10.1007/s00330-020-07403-6 | Yes | Yes | Yes | Yes | Yes | No | No | No |
| 10.1007/s11307-018-1225-8 | Yes | No | Yes | No | Yes | No | No | No |
| 10.1016/j.lfs.2018.07.007 | Yes | Yes | Yes | Yes | Yes | No | No | No |
| 10.1007/s00330-019-06558-1 | Yes | Yes | Yes | Yes | Yes | No | No | No |
| 10.3389/fonc.2020.575422 | Yes | Yes | Yes | Yes | Yes | No | No | No |
| 10.1007/s00330-018-5683-9 | Yes | Yes | Yes | Yes | Yes | No | No | No |
| 10.1007/s00330-019-06572-3 | Yes | Yes | Yes | Yes | Yes | No | No | Yes |
| 10.1007/s11547-017-0838-3 | Yes | Yes | Yes | Yes | Yes | No | No | Yes |
| 10.1002/ctm2.31 | Yes | Yes | Yes | Yes | Yes | No | No | No |
| 10.1007/s00330-020-07673-0 | Yes | Yes | Yes | Yes | Yes | No | No | No |
| 10.1093/jnC-index/djaa017 | Yes | Yes | Yes | Yes | Yes | No | No | No |
| 10.1016/j.ijrobp.2018.04.065 | Yes | Yes | Yes | Yes | Yes | No | No | Yes |
| 10.1136/gutjnl-2018-316407 | No | Yes | Yes | Yes | Yes | No | No | Yes |
| 10.21037/tcr.2019.11.41 | Yes | No | Yes | Yes | Yes | No | No | No |
| 10.1186/s40644-020-00308-z | Yes | Yes | Yes | Yes | Yes | No | No | No |
| 10.1016/j.acra.2019.02.009 | Yes | Yes | Yes | Yes | Yes | No | No | No |
| 10.1088/1361-6560/ab7970 | Yes | No | No | Yes | Yes | No | No | No |
| 10.1109/EMBC44109.2020.9176627 | No | No | No | Yes | Yes | No | No | No |
| 10.1007/s00259-018-4250-6 | Yes | No | Yes | Yes | Yes | No | No | No |
| 10.1007/s00261-019-02117-w | Yes | Yes | Yes | Yes | Yes | No | No | No |
| 10.1148/radiol.2021202363 | Yes | Yes | Yes | Yes | Yes | No | No | Yes |
| 10.1186/s12880-020-00457-4 | Yes | Yes | Yes | Yes | Yes | No | No | No |
| 10.1002/jcp.28650 | Yes | Yes | Yes | Yes | Yes | No | No | No |
| 10.1016/j.ejrad.2019.02.022 | Yes | Yes | Yes | Yes | No | No | No | No |
| 10.1148/radiol.2018172300 | Yes | Yes | Yes | Yes | Yes | No | No | No |
| 10.1007/s00330-018-5539-3 | Yes | Yes | Yes | Yes | Yes | No | No | No |
| 10.1200/JCO.2015.65.9128 | Yes | Yes | Yes | Yes | Yes | No | No | No |
| 10.21147/j.issn.1000-9604.2018.01.05 | Yes | Yes | Yes | Yes | Yes | No | No | No |
| 10.1016/j.acra.2018.01.020 | Yes | Yes | Yes | Yes | Yes | No | No | No |
| 10.3390/cancers13030392 | Yes | Yes | Yes | Yes | Yes | No | Yes | No |
| 10.1016/j.ejrad.2020.109326 | Yes | Yes | Yes | Yes | Yes | No | No | No |
| 10.21037/qims.2019.12.16 | Yes | Yes | Yes | Yes | Yes | No | No | No |
| 10.1002/cam4.2636 | Yes | Yes | Yes | Yes | Yes | Yes | No | No |
| 10.1007/s00432-020-03354-z | Yes | Yes | Yes | Yes | Yes | No | No | No |
| 10.1186/s12967-020-02215-0 | Yes | Yes | Yes | Yes | Yes | No | No | No |
| 10.18632/oncotarget.8919 | Yes | No | Yes | Yes | Yes | No | No | No |
| 10.1016/j.acra.2018.12.019 | Yes | Yes | Yes | Yes | Yes | No | No | No |
| 10.1097/MD.0000000000019251 | Yes | No | Yes | Yes | Yes | No | No | No |
| 10.1007/s00330-018-5802-7 | Yes | Yes | Yes | Yes | Yes | No | No | No |
| 10.1038/s41467-020-18162-9 | Yes | Yes | Yes | Yes | Yes | Yes | No | No |
| 10.1158/1078-0432.CCR-17-1038 | Yes | Yes | Yes | Yes | Yes | No | No | No |
| 10.1007/s00259-017-3855-5 | Yes | No | No | Yes | Yes | No | No | No |
| 10.1186/s12880-019-0392-7 | Yes | No | Yes | Yes | Yes | No | Yes | No |
| 10.1007/s00330-018-5763-x | Yes | Yes | Yes | Yes | Yes | Yes | No | No |
| 10.1245/s10434-020-08974-w | Yes | Yes | Yes | Yes | Yes | No | No | Yes |
| 10.1158/1078-0432.CCR-15-2997 | Yes | No | Yes | Yes | Yes | No | No | No |
| 10.3390/cancers12102978 | Yes | Yes | Yes | Yes | Yes | No | No | No |
| 10.4143/crt.2019.050 | Yes | Yes | Yes | Yes | Yes | No | No | No |
| 10.1007/s00261-020-02502-w | Yes | Yes | Yes | Yes | Yes | Yes | No | No |
| 10.1016/j.ejmp.2019.03.013 | Yes | Yes | Yes | Yes | Yes | No | No | No |
| PMID: 33415015 | Yes | Yes | No | Yes | Yes | No | No | No |
| 10.1016/j.mri.2019.05.003 | Yes | No | Yes | Yes | Yes | No | No | No |
| 10.1038/s41598-019-39651-y | Yes | Yes | Yes | Yes | Yes | No | No | No |
| 10.1016/j.radonc.2019.03.011 | No | No | Yes | Yes | Yes | No | No | No |
| 10.1038/s41598-019-51629-4 | No | Yes | Yes | Yes | Yes | No | Yes | No |
| 10.3389/fonc.2019.01250 | Yes | Yes | Yes | Yes | Yes | No | No | No |
| 10.1016/j.acra.2019.12.007 | Yes | Yes | Yes | Yes | Yes | No | No | No |
| 10.1007/s00384-019-03455-3 | Yes | Yes | Yes | Yes | Yes | No | No | No |
| 10.1007/s00330-017-5146-8 | Yes | Yes | Yes | Yes | Yes | No | No | No |
| 10.1007/s11307-020-01552-2 | Yes | No | Yes | Yes | Yes | No | No | No |
| 10.1245/s10434-019-07300-3 | Yes | Yes | Yes | Yes | Yes | No | No | No |
| 10.3389/fonc.2021.687771 | Yes | Yes | Yes | Yes | Yes | No | No | Yes |
| 10.1038/s41598-022-10175-2 | Yes | No | Yes | Yes | Yes | No | No | No |
| 10.21037/atm.2020.01.107 | Yes | Yes | Yes | Yes | Yes | No | No | No |
| 10.1016/j.radonc.2019.07.033 | Yes | Yes | No | Yes | Yes | No | No | No |
| 10.3389/fonc.2021.671636 | Yes | Yes | Yes | Yes | Yes | No | No | Yes |
| 10.1007/s00330-021-08104-4 | Yes | No | Yes | Yes | Yes | No | No | Yes |
| 10.1007/s12029-019-00291-0 | Yes | Yes | No | Yes | Yes | No | No | No |
| 10.1007/s00261-021-03021-y | Yes | No | Yes | Yes | Yes | No | No | No |
| 10.1016/j.acra.2021.12.016 | Yes | Yes | Yes | Yes | Yes | No | No | No |
| 10.3389/fonc.2020.00459 | Yes | Yes | Yes | Yes | Yes | No | No | No |
| 10.1259/bjr.20211014 | Yes | Yes | Yes | Yes | Yes | No | No | No |
| 10.3389/fonc.2022.828904 | Yes | Yes | Yes | Yes | Yes | No | No | No |
| 10.1038/s41598-021-88831-2 | Yes | Yes | Yes | Yes | Yes | No | No | No |
| 10.1007/s12149-021-01605-8 | Yes | Yes | Yes | Yes | Yes | No | No | No |
| 10.5603/RPOR.a2021.0004 | No | No | Yes | Yes | Yes | No | No | No |
| 10.1007/s00261-020-02710-4 | Yes | Yes | Yes | Yes | Yes | No | No | No |
| 10.1002/jmri.26972 | Yes | Yes | Yes | Yes | Yes | No | No | No |
| 10.1007/s12149-021-01622-7 | Yes | Yes | No | Yes | No | No | No | No |
| 10.3233/XST-210877 | Yes | Yes | Yes | Yes | Yes | No | No | No |
| 10.1016/j.radonc.2020.09.039 | Yes | No | Yes | Yes | Yes | No | No | No |
| 10.3389/fonc.2021.702055 | Yes | Yes | Yes | Yes | Yes | No | No | No |
| 10.1186/s12967-022-03262-5 | Yes | Yes | Yes | Yes | Yes | No | No | No |
| 10.3389/fonc.2020.00457 | Yes | Yes | Yes | Yes | Yes | No | No | No |
| 10.1002/jmri.25969 | Yes | Yes | Yes | Yes | Yes | No | No | No |
| 10.1186/s12967-021-02919-x | Yes | Yes | Yes | Yes | Yes | No | No | No |
| 10.1007/S00261-022-03477-6 | Yes | Yes | Yes | Yes | Yes | No | No | No |
| 10.1186/S12880-022-00773-X | Yes | No | Yes | Yes | Yes | No | No | No |
| 10.1186/S12885-022-09518-Z | Yes | Yes | Yes | Yes | Yes | Yes | No | No |
| 10.3389/FONC.2021.610338 | Yes | Yes | Yes | Yes | Yes | No | No | No |
| 10.1007/S00330-021-08144-W | Yes | Yes | Yes | Yes | Yes | No | No | No |
| 10.1016/j.adro.2020.04.016 | Yes | No | Yes | Yes | No | No | No | No |
| 10.1038/S41416-022-01786-7 | Yes | Yes | Yes | Yes | Yes | No | No | Yes |
| 10.1148/RADIOL.211986 | Yes | Yes | Yes | Yes | Yes | Yes | No | No |
| 10.3389/FONC.2021.614052 | Yes | Yes | Yes | Yes | Yes | No | No | No |
| 10.1038/S41598-021-84816-3 | Yes | No | Yes | Yes | Yes | No | No | No |
| 10.1016/j.acra.2020.10.026 | Yes | No | Yes | Yes | Yes | No | No | No |
| 10.3389/FONC.2022.801743 | Yes | Yes | Yes | Yes | Yes | No | No | No |
| 10.1016/j.acra.2020.09.024 | Yes | Yes | Yes | Yes | Yes | No | No | No |
| 10.3389/fonc.2019.00552 | Yes | Yes | Yes | Yes | Yes | No | No | No |
| 10.3748/wjg.v26.i19.2388 | Yes | Yes | Yes | Yes | Yes | No | No | No |
| 10.1007/S00261-021-03397-X | Yes | Yes | Yes | Yes | Yes | No | No | No |
| 10.3389/FGENE.2022.880093 | No | No | Yes | Yes | Yes | No | No | No |
| 10.1007/S00261-021-03219-0 | Yes | Yes | Yes | Yes | Yes | Yes | No | No |
| 10.1007/s00330-020-07403-6 | Yes | Yes | Yes | Yes | Yes | No | No | No |
| 10.3389/FONC.2020.585767 | Yes | Yes | Yes | Yes | Yes | No | No | No |
| 10.1007/S00330-022-08954-6 | Yes | No | Yes | Yes | Yes | No | No | Yes |
| 10.1002/jmri.25968 | Yes | Yes | Yes | Yes | Yes | Yes | No | No |
| 10.3389/FONC.2022.850774 | Yes | Yes | Yes | Yes | Yes | No | No | No |
| 10.1007/S00330-021-08167-3 | Yes | Yes | Yes | Yes | Yes | No | No | No |
| 10.3390/cancers12071894 | Yes | Yes | Yes | Yes | Yes | No | No | No |
| 10.1016/j.ejrad.2020.109205 | Yes | No | Yes | Yes | Yes | No | No | Yes |
| 10.1007/s00330-021-07877-y | Yes | Yes | Yes | Yes | Yes | No | No | No |
| 10.3389/fonc.2021.697497 | Yes | Yes | Yes | Yes | Yes | No | No | No |
| 10.3233/XST-190614 | Yes | Yes | Yes | Yes | Yes | No | No | Yes |
| 10.3389/fonc.2021.671354 | Yes | Yes | Yes | Yes | Yes | No | No | No |
| 10.1016/j.acra.2021.11.024 | Yes | Yes | Yes | Yes | Yes | Yes | No | No |
| 10.3748/wjg.v27.i33.5610 | Yes | Yes | Yes | Yes | Yes | No | No | No |
| 10.3390/cancers11111680 | No | No | Yes | Yes | No | Yes | No | No |
| 10.1093/bjs/znab191 | Yes | No | Yes | Yes | Yes | Yes | No | Yes |
| 10.1007/s00330-020-06968-6 | Yes | No | Yes | Yes | Yes | No | No | Yes |
| 10.3748/wjg.v26.i33.5008 | Yes | No | Yes | Yes | Yes | No | No | No |
| 10.5152/dir.2021.19677 | Yes | Yes | Yes | Yes | Yes | No | No | No |
| PMCID: PMC6682712 | Yes | No | Yes | Yes | Yes | No | No | No |
| 10.1148/rycan.2020190084 | Yes | No | Yes | Yes | No | No | No | No |
| 10.3389/fonc.2020.00604 | Yes | Yes | Yes | Yes | Yes | No | No | No |
| 10.1158/1078-0432.CCR-15-2997 | Yes | Yes | Yes | Yes | Yes | Yes | No | No |
| 10.1002/jmri.28108 | Yes | Yes | Yes | Yes | Yes | No | No | No |
| 10.1186/s13014-022-02053-y | Yes | Yes | Yes | Yes | Yes | Yes | No | Yes |
| 10.1007/s00261-021-03311-5 | No | Yes | Yes | Yes | Yes | No | No | Yes |
| 10.1007/s00330-020-07192-y | Yes | No | Yes | Yes | Yes | No | No | No |
| 10.1007/s00261-022-03453-0 | Yes | Yes | Yes | Yes | Yes | No | No | No |
| 10.1002/mp.14896 | Yes | No | Yes | Yes | Yes | No | No | Yes |
| 10.18632/oncotarget.13855 | No | Yes | Yes | Yes | Yes | No | No | Yes |
| 10.1002/MP.15001 | Yes | Yes | Yes | Yes | Yes | No | No | No |
| 10.1016/j.acra.2022.02.00 | No | Yes | Yes | Yes | Yes | No | No | Yes |
| 10.3389/fonc.2020.574337 | Yes | Yes | No | Yes | Yes | Yes | No | No |
| 10.3389/fonc.2020.00457 | Yes | Yes | Yes | Yes | Yes | No | No | No |
| 10.3389/fonc.2022.831712 | No | No | Yes | Yes | Yes | No | No | Yes |
| 10.1016/J.RADONC.2021.07.004 | Yes | Yes | Yes | Yes | Yes | No | No | No |
| 10.1007/s00261-021-03137-1 | No | Yes | Yes | Yes | Yes | No | No | No |
| 10.3390/cancers14123004 | Yes | Yes | Yes | Yes | Yes | No | No | Yes |
| 10.3390/cancers14081895 | Yes | Yes | Yes | Yes | Yes | No | No | No |
| 10.1186/S12880-022-00813-6 | Yes | Yes | Yes | Yes | Yes | No | No | No |
| 10.1007/S00261-020-02863-2 | Yes | Yes | Yes | Yes | Yes | No | No | No |
| 10.1186/S13014-022-02048-9 | No | No | Yes | Yes | Yes | No | No | Yes |
| 10.3389/FONC.2022.861892 | Yes | Yes | Yes | Yes | Yes | No | No | Yes |
| 10.3389/FONC.2022.843991 | Yes | No | Yes | Yes | Yes | No | No | No |
| 10.3390/cancers14092231 | No | No | Yes | Yes | Yes | No | No | No |
| 10.4254/wjh.v14.i1.244 | Yes | Yes | Yes | Yes | Yes | No | No | No |
| 10.3389/FONC.2021.694102 | Yes | Yes | Yes | Yes | Yes | No | No | No |
| 10.1007/S11307-022-01730-4 | Yes | Yes | Yes | Yes | Yes | No | No | No |
| 10.1007/s00261-022-03572-8 | Yes | Yes | Yes | Yes | Yes | Yes | No | Yes |
| 10.21873/invivo.12126 | Yes | No | Yes | Yes | Yes | No | No | No |
| 10.1016/J.TRANON.2022.101352 | Yes | Yes | Yes | Yes | Yes | No | No | No |
| 10.1016/J.EJRAD.2021.110065 | No | Yes | Yes | Yes | Yes | No | No | Yes |
| 10.3389/FONC.2021.710248 | Yes | Yes | Yes | Yes | Yes | No | No | No |
| 10.3389/fonc.2021.666786 | Yes | Yes | Yes | Yes | Yes | No | No | Yes |
| 10.3390/cancers14051110 | Yes | Yes | Yes | Yes | Yes | No | No | No |
| 10.21147/j.issn.1000-9604.2022.01.04 | Yes | Yes | Yes | Yes | Yes | No | No | Yes |
| 10.1111/1754-9485.13044 | Yes | Yes | Yes | Yes | Yes | No | No | No |
| 10.3389/fonc.2021.644933 | Yes | Yes | Yes | Yes | Yes | No | No | No |
| 10.1016/j.ebiom.2021.103442 | Yes | No | No | Yes | Yes | No | No | Yes |
| 10.1007/s00330-022-08952-8 | Yes | Yes | Yes | Yes | Yes | No | No | Yes |
| 10.3389/fonc.2020.595012 | Yes | Yes | Yes | Yes | Yes | No | No | No |
| 10.1016/j.ejmp.2021.03.038 | Yes | Yes | Yes | No | Yes | No | No | No |
| 10.1186/s13014-019-1246-8 | Yes | No | Yes | Yes | Yes | No | No | No |
| 10.1186/s12885-022-09584-3 | Yes | Yes | Yes | Yes | Yes | No | No | No |
| 10.1007/s00261-020-02846-3 | Yes | Yes | Yes | Yes | Yes | No | No | No |
| 10.21037/atm-20-7673 | Yes | Yes | Yes | Yes | Yes | No | No | No |
| 10.2147/CMAR.S295317 | Yes | Yes | Yes | Yes | Yes | No | No | Yes |
| 10.1007/s11547-021-01421-0 | Yes | Yes | Yes | Yes | Yes | No | No | No |
| 10.1186/s12880-021-00560-0 | Yes | Yes | Yes | Yes | Yes | No | No | No |
| 10.1155/2021/4520450 | No | No | No | No | Yes | No | No | No |
| 10.3390/cancers14041079 | Yes | Yes | Yes | Yes | Yes | No | No | Yes |
| 10.1007/s11596-020-2298-6 | Yes | Yes | Yes | Yes | Yes | No | No | No |
| 10.3389/fonc.2021.774413 | Yes | Yes | Yes | Yes | Yes | No | No | No |
| 10.1186/s40644-021-00408-4 | Yes | Yes | Yes | Yes | Yes | No | No | No |
| 10.1007/s00261-020-02733-x | Yes | Yes | Yes | Yes | Yes | No | No | No |
| 10.1007/s00261-022-03534-0 | Yes | Yes | Yes | Yes | Yes | No | No | No |
| 10.3389/fonc.2021.620945 | Yes | Yes | Yes | Yes | Yes | No | No | No |
